# IL-10-providing B cells govern pro-inflammatory activity of macrophages and microglia in CNS autoimmunity

**DOI:** 10.1007/s00401-023-02552-6

**Published:** 2023-03-01

**Authors:** Anastasia Geladaris, Silke Häusser-Kinzel, Roxanne Pretzsch, Nitzan Nissimov, Klaus Lehmann-Horn, Darius Häusler, Martin S. Weber

**Affiliations:** 1 Institute of Neuropathology, University Medical Centre Göttingen, Göttingen, Germany; 2 Fraunhofer-Institute for Translational Medicine and Pharmacology ITMP, Göttingen, Germany; 3 Department of Neurology, University Medical Centre Göttingen, Göttingen, Germany; 4 Department of Neurosurgery, Charité-Universitätsmedizin Berlin Corporate Member of Freie Universität Berlin and Humboldt-Universität zu Berlin, Berlin, Germany; 5 Department of Neurology, School of Medicine, Technical University of Munich, Munich, Germany

**Keywords:** Regulatory B cells, Interleukin-10, Multiple sclerosis, Experimental autoimmune encephalomyelitis, Myeloid cells, Microglia

## Abstract

B cells contribute to chronic inflammatory conditions as source of antibody-secreting plasma cells and as antigen-presenting cells activating T cells, making anti-CD20-mediated B cell depletion a widely used therapeutic option. B cells or B cell subsets may, however, exert regulatory effects, while to date, the immunological and/or clinical impact of these observations remained unclear. We found that in multiple sclerosis (MS) patients, B cells contain regulatory features and that their removal enhanced activity of monocytes. Using a co-culture system, we identified B cell-provided interleukin (IL)-10 as key factor in controlling pro-inflammatory activity of peripheral myeloid cells as well as microglia. Depleting B cells via anti-CD20 in a mouse model of MS unleashed the activity of myeloid cells and microglia and accelerated disease severity; in contrast, adoptive transfer of IL-10-providing B cells restored in vivo control of central nervous system (CNS) macrophages and microglia and reversed clinical exacerbation. These findings suggest that B cells exert meaningful regulatory properties, which should be considered when designing novel B cell-directed agents.

**Supplementary Information:**

The online version contains supplementary material available at 10.1007/s00401-023-02552-6.

## Introduction

In patients with multiple sclerosis (MS), anti-CD20 antibody-mediated depletion of B cells enables an excellent therapeutic control of acute relapses [1, 15, 29]. It is assumed that anti-CD20´s mode of action includes abrogation of both antigen presentation and provision of pro-inflammatory cytokines by B cells [19], which results in reduced T cell activation. However, besides the development of relapses, most people with MS accumulate permanent disability over time by an underlying, smouldering process within the central nervous system (CNS). This process is not fully understood, but likely initiates relatively early and subtly in the disease course, when acute relapses are clinically more apparent and surfaces as chronic progression when the parenchymal and functional reserve of the CNS is exhausted. Pathophysiologically, chronic progression is attributed to a CNS-intrinsic interplay of CNS-established lymphocytes including T cells, B cells, monocytes, and macrophages within meningeal areas and chronic activation of CNS-resident cells such as microglia und astrocytes in the parenchyma [6, 18]. What the exact functional role of B and plasma cells is in this process is unclear. In MS, B cells are found in the parenchyma, but primarily in the cerebrospinal fluid (CSF), perivascular locations and meninges [5, 16, 23, 25, 38]. Especially during periods of active disease, the CSF of patients with MS contains molecules that strongly fosters B cell recruitment and activity [7, 27] and is enriched in B and plasma cells [33, 40]. These cells are the source of intrathecally produced immunoglobulin (Ig)G in more than 95% of all patients with MS [13, 31], suggesting a continuous presence and activation of B cells within the CNS. Hence, it is not surprising that many studies link accelerated disease progression to the presence of B cells in the CNS compartment [4, 24, 26]. However, besides pro-inflammatory properties, B and plasma cells exhibit various regulatory features [10]. In the context of experimental CNS autoimmunity both physical interactions [34, 37, 42] as well as the secretion of anti-inflammatory cytokines e.g. interleukin (IL)-10 have been shown to be relevant for disease regulation [2, 11, 30, 35, 39] and IL-10-expressing plasma cells have been found in CNS lesions of patients with MS [25, 32] and mice with experimental autoimmune encephalomyelitis (EAE) [28]. Moreover, B cells are assumed to control pro-inflammatory activity of other peripheral immune cells [12, 20, 30], even though IL-10-secreting B cells have been reported to be decreased in number [17] and impaired in function [8] in the blood of people with MS. Accordingly, B cells most likely exert both pro- and anti-inflammatory properties in MS. Based on this scenario, and the relatively uncritical use of anti-CD20 mediated pan B cell depletion in MS, but also numerous other chronic inflammatory conditions, we set out to study the functional and possibly clinical impact of B cell regulation in MS and its animal model EAE.

## Material and methods

### Human samples

After written informed consent was obtained, healthy donors and patients with clinically isolated syndrome, relapsing–remitting or secondary progressive MS were enrolled for our study at the Technical University of Munich, Germany and the University Medical Centre Göttingen, Germany. The study protocol was approved by the ethics committees of the University Medical Centre Göttingen (#03/04/14 and #12/6/21) and the Technical University of Munich (#19/09/10). For detailed characteristics of the study participants, please see Supplementary Tables 1 and 2 (online resource). Patients designated for anti-CD20 antibody treatment had received no corticosteroids or any immunosuppressive or immunomodulatory therapy (other than Rituximab) within two months prior to enrolment. Rituximab was administered at a dose of 1000 mg on days 1 and 15. Blood samples were collected during routine clinical assessment at two time points: before anti-CD20 antibody treatment and 11–28 weeks thereafter when B cells were still absent (confirmed by flow cytometry). Detailed descriptions of the study materials and methods are provided in the sections below.

### Mice

Wild-type C57BL/6J mice were purchased from Charles River (Sulzfeld, Germany). MOG p35-55 TCR transgenic 2D2 mice were kindly provided by Dr. Kuchroo (Boston, USA). CD20 KO mice were generated and provided by Genentech. IL-10 KO mice were purchased from Jackson Laboratory. All animal experiments were carried out in accordance with the guidelines of the Central Department for Animal Experiments, University Medical Centre, Göttingen and approved by the Office for Consumer Protection and Food Safety of the State of Lower Saxony (protocol number 33.9-42502-04-15/1804, 33.9-42502-04-19/3244 and 33.9-42502-04-20/3489).

### Isolation of human and murine leukocytes

Peripheral blood mononuclear cells (PBMC) from human study subjects were isolated via Ficoll gradient centrifugation. Human total B cells (purity > 98%) and CD27-negative and CD27-positive B cells were isolated from PBMC using the B cell Isolation Kit II and the Memory B cell Isolation Kit (both Miltenyi Biotec), respectively. Human CD14-positive cells were isolated from PBMC by magnetic-activated cell sorting (MACS) using anti-CD14 MicroBeads (Miltenyi Biotec; purity > 90%). Single cell suspensions of murine lymphoid tissues were generated by gentle dissection and passing through 70 µm cell strainer (Greiner Bio-One). Brain and spinal cord tissues were isolated from mice upon perfusion with phosphate-buffered saline (PBS) and were dissociated to single cells using the Multi Tissue Dissociation Kit (Miltenyi Biotec). Murine B cells (purity > 95%) were isolated from spleens by MACS using anti-CD19 MicroBeads (Miltenyi Biotec).

### In vitro culture of human B cells

3.5 × 10^5^ MACS-purified B cells of patients with MS and healthy controls, as well as CD27-positive and CD27-negative B cells from healthy individuals, were plated in 96-well u-bottom plates and stimulated with either 4 µg/ml CpG or with 40 µg/ml affiniPure F(ab')2 fragment rabbit anti-human IgM (Fc5 Fragment Specific, Jackson Immuno Research) and 1 µg/ml recombinant human CD40L (R&Dsystem). Unstimulated samples served as controls. After 48 h of culture, supernatants were collected, and secreted IL-6 and IL-10 were determined by enzyme-linked immunosorbent assay (ELISA).

### Enzyme-linked immuno spot assay (ELISpot) for analysis of TNF-producing human myeloid cells

3,000 MACS-isolated CD14-positive cells/well were plated in triplicates in TNF capture antibody-coated (human TNFα ELISPOT Antibody Pair, Millipore) Multi-Screen Filter Plates (Millipore) and stimulated with lipopolysaccharide (LPS; *E. coli* O111:B4; Sigma) for 18 h. Plates were washed and incubated successively with TNF detection antibody, streptavidin–alkaline phosphatase and BCIP/NBT substrate (all human TNF-α ELISPOT Antibody Pair, Millipore). Plates were analysed with an automated imaging system and software (AID ELISpot reader and software, Autoimmun Diagnostika or AELVIS ELISpot reader and software, Stefan Badur Electronic GmbH & Co. KG).

### Generation of bone marrow‑derived myeloid cells

To generate bone marrow-derived myeloid cells (BMDM), bone marrow isolated from hind limbs of C57Bl/6J mice was cultured at 37 °C and 5% CO_2_ for seven days in medium containing 30% conditioned L929 cell supernatant (DMEM, 30% L929 supernatant, 10% foetal calf serum, 5% horse serum, 50 U/ml penicillin, 50 μg/ml streptomycin, 0.05 mM β-mercaptoethanol). Adherent BMDM were detached using 2.5% trypsin (Pan Biotech) and 0.4 mg DNAse I (Roche) and harvested using cell scrapers. Cultures contained > 95% myeloid cells verified by flow cytometry.

### Generation of primary microglia

To generate primary microglia, brain cells of new-born to two-day-old C57BL/6J mice were isolated enzymatically using 2.5% trypsin (Pan Biotech) and 0.4 mg DNAse I (Roche). First, a mixed glial cell culture was generated by seeding the cells in DMEM containing 10% foetal calf serum, 1% GlutaMax™, 100 U/ml penicillin, and 100 µg/ml streptomycin and cultivating them at 37° C and 5% CO_2_ until confluency was reached. To obtain enriched microglia cultures, cells were thereafter stimulated for five days with medium containing 30% conditioned L929 cell supernatant (DMEM, 30% L929 supernatant, 10% foetal calf serum, 100 U/ml penicillin, and 100 µg/ml streptomycin). Primary microglia were harvested by gentle shaking at 90 rpm for 30 min at 37 °C to separate microglial cells from other glia cell. Cultures contained > 97% microglial cells verified by flow cytometry.

### Isolation and stimulation of murine B cells, generation of B cell supernatant and neutralization of IL-10

Splenic B cells were isolated from C57Bl/6J mice using MACS (mouse anti-CD19 MicroBeads; BioLegend) and purity (> 95%) was evaluated by flow cytometry. Cytokine secretion was induced by stimulation with 5 µg/ml LPS (*E. coli* O111:B4; Sigma). For the co-culture experiments, B cells were harvested after 24 h and washed thoroughly to remove LPS. For the experiments with soluble B cell products, B cell supernatants were collected after 48 h of culture and LPS was removed using Pierce™ high capacity endotoxin removal spin columns (ThermoFisher) according to manufacturer’s instructions. Where indicated, IL-10 was neutralized by adding 1 µg/ml anti-IL-10 antibody (clone: JES5-2A5; BioXcell) or isotype control antibody (clone: TNP6A7; BioXcell) to the B cell supernatant 20 min before further use.

### Culture of bone marrow-derived myeloid cells and microglia with B cells or their soluble products

BMDM were plated at a density of 0.5 × 10^5^ cells/well into 96-well flat-bottom plates and stimulated with 100 ng/ml LPS. Primary microglia were plated at a density of 2 × 10^5^ cells/well into 12-well plates and stimulated with 1 ng/ml LPS. For co-culture with B cells, 2 × 10^5^ pre-stimulated B cells were added to BMDM or microglia and incubated for 24–48 h. Where indicated, cells were separated by a transwell system (Corning) to inhibit cellular contact between BMDM or microglia and B cells. In brief, BMDM or microglia were cultured on the bottom of a 96-well plate and pre-stimulated B cells were seeded on top into a membrane-insert with 0.4 µm pores that allows exchange of soluble factors, but prevents cellular transmigration. For culture with soluble B cell factors, supernatant of LPS-stimulated B cells, where indicated neutralized for IL-10, was added to BMDM or microglia in a ratio of 1:1. After 24–48 h, cell supernatants were harvested for ELISA, and BMDM/microglia were detached using 0.05% trypsin and 0.02% ethylenediaminetetraacetic acid (EDTA; *w*/*v*) in PBS for analysis by flow cytometry.

### Assessment of T cell proliferation and differentiation in vitro

Adherent BMDM or microglia were detached using 0.05% trypsin and 0.02% EDTA (*w*/*v*) in PBS. BMDM (0.5 × 10^5^ cells/well) or microglia (0.3 × 10^5^ cells/well) were plated into 96-well flat-bottom plates and stimulated with 100 ng/ml or 1 ng/ml LPS, respectively. Where indicated, rIL-10 (1 ng/ml), complete B cell supernatant or B cell supernatant neutralized for IL-10 were added additionally. After 24 h, BMDM/microglia were washed twice and 0.5 × 10^5^ MACS-purified (Pan T cell Isolation Kit, Miltenyi, Bergisch Gladbach, Germany) carboxyfluorescein succinimidyl ester (CFSE)-stained (CFSE Cell Division Tracker Kit, BioLegend) or unstained T cells from 2D2 mice were added per well. 72 h after co-culture in the presence of MOG p35-55, T cell proliferation and differentiation were evaluated by flow cytometry and/or ELISA.

### EAE induction and scoring

As indicated in the respective figure legend, female wild-type mice were immunized subcutaneously with 50 or 75 µg MOG p35-55 MEVGWYRSPFSRVVHLYRNGK (Auspep) or 75 μg MOG protein 1–117 (GenScript Biotech) emulsified in complete Freund’s adjuvant (Sigma-Aldrich) containing 250 µg inactivated *Mycobacterium tuberculosis* H37 Ra (BD Bioscience) followed by intraperitoneal injections of 200 ng of *Bordetella pertussis* toxin (Sigma-Aldrich) on the day of immunization and two days thereafter. EAE severity was assessed daily and scored on a scale from 0 to 5 as follows: 0 = no clinical signs; 1.0 = tail paralysis; 2.0 = hindlimb paresis; 3.0 = severe hindlimb paresis; 4.0 = paralysis of both hindlimbs; 4.5 = hindlimb paralysis and beginning forelimb paresis; and 5.0 = moribund/death.

### Anti-CD20 treatment

Mice received weekly intraperitoneal injections of 0.2 mg murine monoclonal anti-CD20 antibody (Clone 5D2; IgG2a) or monoclonal anti-HIV-1 (Clone: gp120; IgG2a) control antibody (both provided by Genentech) starting three weeks prior to immunization or at the indicated time points.

### Adoptive B cell transfer

C57BL/6J recipient mice were depleted of B cells by weekly intraperitoneal injections of 0.2 mg murine anti-CD20 antibody during the whole experiment. 10 × 10^6^ CD20KO or CD20KO/IL10KO B cells were intravenously injected into recipient mice once a week for three consecutive weeks, starting three weeks after the first anti-CD20 antibody injection. Six weeks after the last B cell transfer, recipient mice were immunized with MOG p35-55 and clinical symptoms were monitored for five weeks.

### Detection of anti-MOG antibodies

96-well plates were coated with 10 μg/ml MOG protein 1–117 in PBS overnight. Thereafter, diluted serum samples were incubated for two hours. After washing, plate-bound antibodies were detected with horseradish peroxidase-conjugated anti-mouse IgG, directed against the Fc part of the bound antibodies. Absorbance was measured at 450 nm with subtraction of a 540 nm reference wavelength on the iMark Microplate Reader.

### Histology and immunohistochemistry

Mice were transcardially perfused with PBS followed by 4% paraformaldehyde (PFA) and tissue was paraffin embedded. One-micrometre thick slices were stained with haematoxylin and eosin and Luxol fast blue/periodic acid-Schiff. T cells, plasma cells, macrophages and microglia were detected by immunohistochemistry with an avidin–biotin technique using antibodies specific for CD3 (SP7; DCS Innovative Diagnostik-Systeme), IgG (polyclonal; Merck), Mac-3 (M3/84; BD Biosciences) and Iba1 (polyclonal; Fujifilm), respectively. Histological sections were captured using a digital camera (DP71; Olympus Europa) mounted on a light microscope (BX51; Olympus Europa). The percentage of demyelinated white matter was calculated using cellSens Dimension software (Olympus Europa). Inflammatory cells were quantified at × 400 magnification using an ocular counting grid and are shown as cells/mm^2^. At least eight spinal cord cross sections per animal were taken for each analysis.

### Enzyme-linked immunosorbent assays (ELISA)

The production of human IL-6 and IL-10 was measured using ELISA MAX Standard Set (BioLegend). The production of murine CCL2, IL-4, IL-6, IFN-γ, IL-17, TNF-α, and granulocyte–macrophage colony-stimulating factor was measured using ELISA MAX Standard Set kits (BioLegend). Murine IL-2, IL-10, IL-12, CCL3, CCL5 and transforming growth factor-beta production were measured using DuoSet ELISA kits (R&D Systems). Absorbance was determined at 450 nm with subtraction of a 540 nm reference wavelength on iMark™ microplate reader (Bio-Rad laboratories Inc.).

### Flow cytometry of human and murine samples

Human PBMC were stained for CD19 (HIB19; BioLegend), CD14 (M5E2; BD Bioscience) and MHC class II (G46-6; BD Bioscience). Composition of murine immune cells was analysed using the following antibodies: CD3 (145-2C11; BioLegend), CD19 (6D5; BioLegend), CD20 (SA275A11; BioLegend), CD11b (M1/70; BioLegend), CD11c (N418; BioLegend), CD45 (30-F11; BioLegend), Ly6C (HK1.4; BioLegend) and Ly6G (1A8: BioLegend). B cell maturation was analysed using the following antibodies: CD19 (6D5; BioLegend), CD21 (7G6; BD Bioscience), CD23 (B3B4; BD Bioscience), CD93 (AA4.1; BioLegend), CD45R/B220 (RA3-6B2; BioLegend), IgD (11-26c.2a; BioLegend) and IgM (AF6-78; BD Bioscience). Monocyte, macrophage and microglia activation, differentiation and molecules involved in antigen presentation were determined using: CD40 (3/23; BD Bioscience), CD68 (FA-11; BioLegend), CD69 (H1.2F3; BioLegend), CD80 (16-10A1; BioLegend), CD86 (GL-1; BioLegend), MHCII (AF6-120.1; BioLegend) and PD-L1 (MIH5; eBioscience). Fc receptors were blocked using monoclonal antibody specific for murine or human CD16/ CD32 (Murine TruStain FcX; Human TruStain FcX; BioLegend), respectively. Dead cells were stained with the Zombie Fixable Viability™ Kit (BioLegend). Samples were acquired on a BD LSR Fortessa (BD Bioscience). All data evaluation was performed using FlowJo software (FlowJo LLC, Ashland, USA).

### Cell viability

The WST-1 cell assay (Roche) was used to determine cell viability. After culture, cells received fresh medium containing WST-1 reagent and were incubated at 37° C, 5% CO_2_ for three hours. Absorbance was determined at 450 nm with subtraction of a 655 nm reference wavelength on iMark™ microplate reader (Bio-Rad laboratories Inc.).

### Statistical analysis

Statistics were calculated using GraphPad Prism 6. For the analysis of ex vivo experiments, Gauss distribution was tested via Shapiro–Wilk normality test if *n* > 6; for experiments with *n* ≤ 6, a non-Gauss distribution was assumed. For the analysis of in vitro experiments, Gauss distribution was assumed. The respective statistical comparisons used are indicated in the figure legends.

## Results

### Both naïve and memory B cells from healthy subjects and patients with MS can produce anti-inflammatory IL-10

**Fig. 1:**
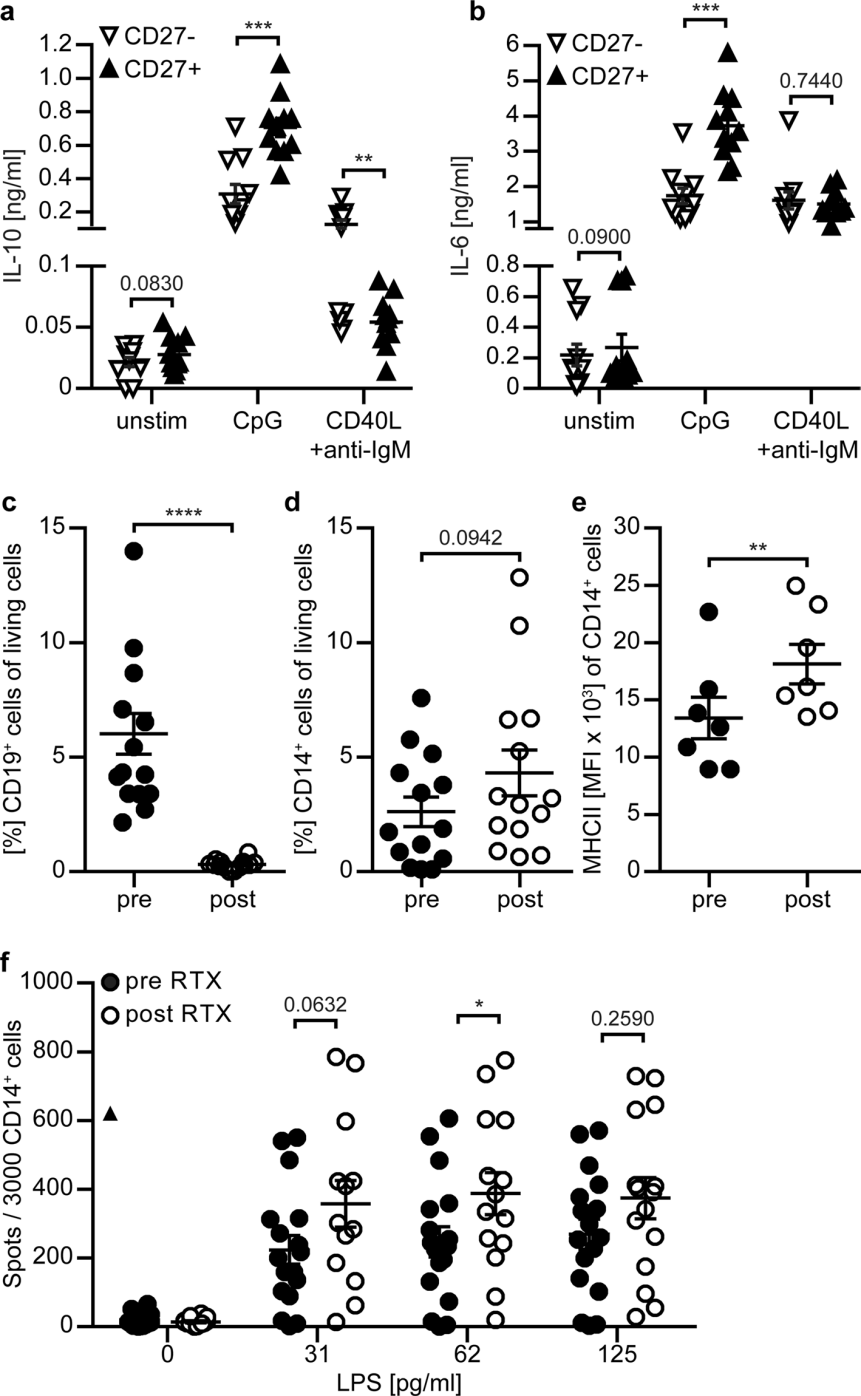
Human B cells secrete relevant amounts of IL-10 and B cell depletion is associated with an enhanced activation of blood myeloid cells. **a**, **b** CD27^−^ and CD27^+^ B cells isolated from peripheral blood mononuclear cells (PBMC) of healthy donors (*n* = 11) were cultured without stimulation (unstim), stimulated with CpG (4 µg/ml) or stimulated with CD40 ligand (CD40L; 1 µg/ml) and anti-IgM antibody (40 µg/ml) for 48 h. Concentrations of secreted **a** IL-10 and **b** IL-6 were determined using ELISA. Frequency of **c** CD19^+^ B cells (*n* = 14), **d** CD14^+^ cells (*n* = 14) and **e** mean fluorescence intensity (MFI) of MHC class II (MHCII) on CD14^+^ cells (data points were included if CD14 frequency > 1% in (**d**); *n* = 7) was determined in PBMC of patients with multiple sclerosis (MS) before (pre) and after (post) anti-CD20 antibody treatment. **f** CD14^+^ cells were isolated from PBMC of patients with MS (*n* = 17) before (pre RTX) and 11–28 weeks after anti-CD20-mediated B cell depletion (post RTX) and stimulated with LPS for 18 h. Number of TNF-α producing CD14^+^ cells determined by ELISpot. The mean ± standard error of the mean is indicated in all graphs. Asterisks indicate significant difference calculated using paired two-tailed *t*-test (*P < 0.05, **P ≤ 0.01, ***P ≤ 0.001)

In order to corroborate regulatory properties of B cells, we determined the cytokine profile of human B cells stimulated via their B cell receptor (BCR), or alternatively via toll-like receptor (TLR) engagement. Moreover, we investigated whether certain B cell subsets preferentially produce anti- versus pro-inflammatory cytokines. For this purpose, blood-derived CD27-negative naïve and CD27-positive memory B cells were isolated from healthy volunteers. Purified B cells were stimulated using anti-IgM antibodies in combination with CD40 ligand (CD40L) or unmethylated cytosine–guanine dinucleotide (CpG), the ligand of TLR9. As hallmark pro- and anti-inflammatory cytokines, B cellular production of IL-6 or IL-10 was assessed. Both stimulation regimens enhanced the production of either cytokine when compared to unstimulated B cells. However, unspecific TLR stimulation raised the release of both pro-inflammatory IL-6 and anti-inflammatory IL-10 with a relatively stronger effect on memory B cells. In contrast, BCR engagement preferentially upregulated the production of IL-6 by naïve and memory B cells, while anti-IgM/CD40L selectively increased the secretion of IL-10 by CD27-negative naïve B cells (Fig. 1a, b; Supplementary Fig. 1a, online resource). In an approach to translate this observation towards MS and B cell-directed therapy, we compared these results to B cells isolated from patients with MS and observed an identical cytokine response pattern to either stimulation (Supplementary Fig. 1b–d, online resource). The meaning of these findings is two-fold; first, regulatory IL-10 production cannot be pinpointed to a specific B cell subset or phenotype, and instead, the context of stimulation determines whether a B cell responds in a regulatory manner. Second and importantly, when compared to healthy controls, these regulatory B cell properties appear to be largely preserved in patients with MS.

### In patients with MS, anti-CD20-mediated depletion of B cells is associated with an enhanced activation of blood myeloid cells

Having substantiated that B cell regulation exists in patients with MS, we next studied whether unselective removal of B cells via anti-CD20 antibodies may cause a detectable loss of these regulatory B cell properties. Specifically, we investigated whether B cell-depleting anti-CD20 may have an effect on the phenotype and frequency of circulating CD14-positive monocytes based on a reported bidirectional interplay of B cells and myeloid antigen-presenting cells (APC) [21]. Accordingly, we collected and analysed blood samples of patients longitudinally assigned to receive anti-CD20 antibody rituximab (RTX): first, before treatment initiation (pre) and second, 12–28 weeks thereafter (post) when B cells were absent (Fig. 1c). As indicated in Fig. 1d, e, monocytes remained relatively stable in their frequency, while the expression of major histocompatibility complex (MHC) class II was significantly enhanced upon therapeutic removal of B cells. Moreover, blood monocytes showed a significant increase in the frequency of cells secreting pro-inflammatory tumour necrosis factor alpha (TNF-α Fig. 1f). In conjunction, these findings suggest that blood myeloid cells gain pro-inflammatory function when B cells are removed unselectively and establish that pan B cell depletion via anti-CD20 collaterally abrogates pre-existing B cell regulatory properties in patients with MS.

### B cells control pro-inflammatory activation and differentiation of both peripheral myeloid APC as well as microglia

**Fig. 2:**
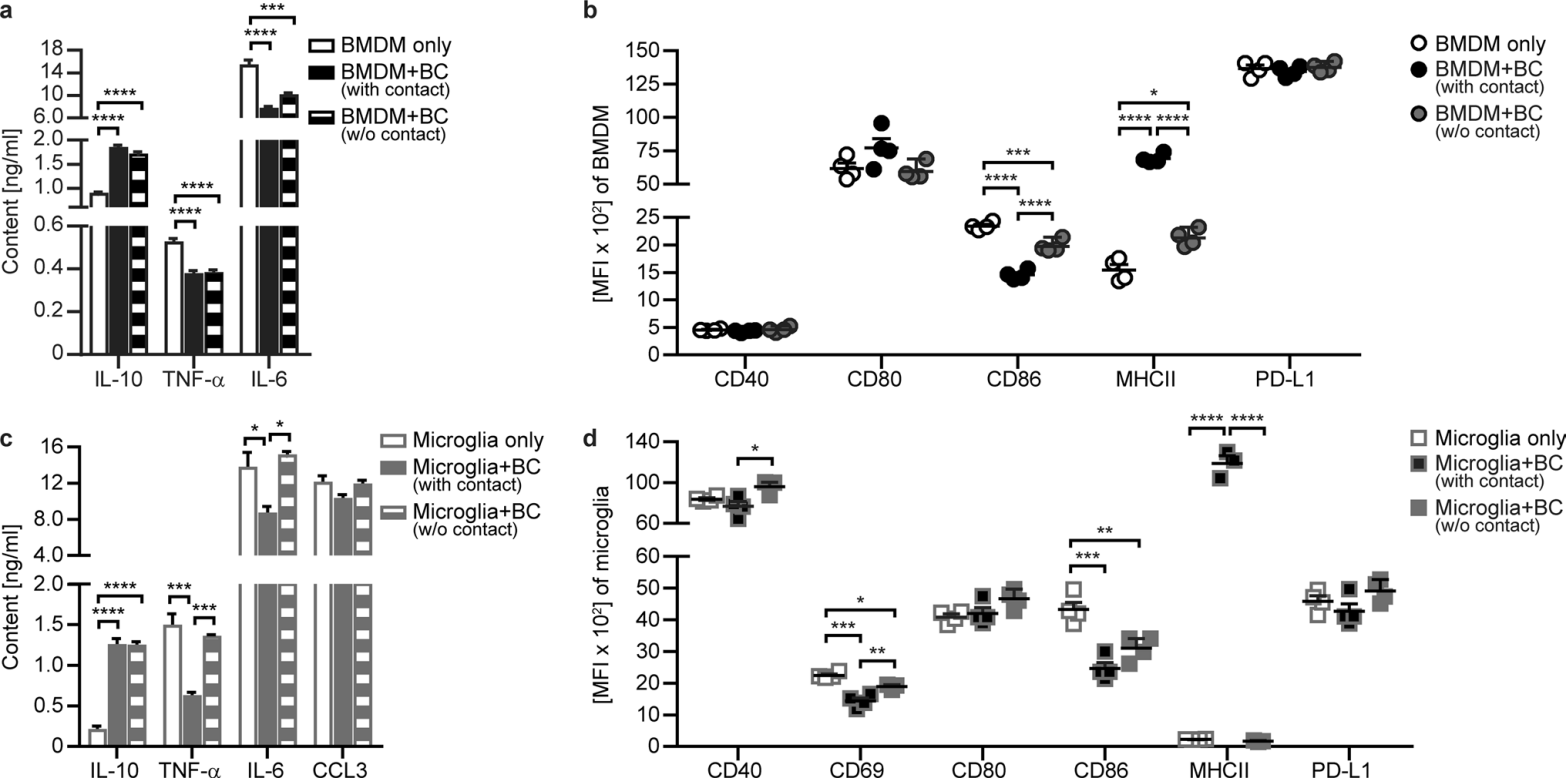
B cells lower the secretion of pro-inflammatory cytokines by myeloid cells and microglia and affect their phenotype. Pre-activated splenic B cells purified from C57BL/6J mice were co-cultured with **a**, **b** bone marrow-derived myeloid cells (BMDM) for 48 h or **c**, **d** primary microglia for 24 h in the presence of LPS. Cell populations were either in direct cell contact (BMDM/Microglia + BC (with contact)) or separated by a fluid-permeable membrane that prevents cellular interactions (BMDM/Microglia + BC (w/o contact)). After co-culture, **a**, **c** cytokine concentrations were determined by ELISA (*n* = 4–8 wells/condition) and **b**, **d** BMDM and microglia were analysed for activation and expression of molecules involved in antigen presentation shown as mean fluorescence intensity (MFI) using flow cytometry (*n* = 4 wells/condition). The mean ± standard error of the mean is indicated in all graphs. Data sets are representative of at least three independent experiments. Asterisks indicate significant differences calculated using one-way analysis of variance corrected by Holm-Sidak (*P ≤ 0.05, **P ≤ *0*.01, ***P ≤ *0*.001, ****P ≤ 0.0001)

These observations generated in humans suggested that regulatory B cell properties have the ability to control the activity of myeloid cells, such as blood monocytes and microglia. In order to dissect mechanistically how B cells may exert regulatory functions on peripheral and CNS-located APC and hence control CNS inflammation, we set up an in vitro assay co-culturing murine B cells with bone marrow-derived myeloid cells (BMDM) or primary microglia. In a first set of experiments, we cultured BMDM together with B cells either in direct cell–cell contact or separated by a fluid-permeable membrane. Using this setting, we observed that in the presence of B cells and independent of cellular contact, secreted IL-10 was increased in the co-culture supernatants of BMDM and B cells, whereas the concentration of pro-inflammatory TNF-α and IL-6 was simultaneously decreased (Fig. 2a). Furthermore, BMDM showed an enhanced expression of MHC class II and a decreased expression of CD86 upon co-culture with B cells, while CD40, CD80 and programmed death-ligand 1 (PD-L1) remained unaltered (Fig. 2b). Of note, these changes in the expression of surface molecules were pronounced when cells were in direct contact. Using the same experimental setting for co-cultures of microglia with B cells, we similarly observed IL-10 to be significantly increased in the culture supernatants when B cells were present independent of direct contact, while the concentrations of pro-inflammatory TNF-α and IL-6 were only reduced when cellular interactions with B cells were not restricted (Fig. 2c). Regarding the expression of surface molecules, co-culture with B cells downregulated CD69 and CD86 on microglia significantly independent of cellular contact, while the expression of CD80 and PD-L1 was unaffected (Fig. 2d). Of note, MHC class II on microglia was highly upregulated when they were in direct cell–cell contact with B cells (Fig. 2d). In conjunction, these data establish that B cells can shape the phenotype and secretory profile of both peripheral myeloid APC as well as CNS microglia. These findings furthermore suggest that the majority of these B cell properties is mediated by soluble factors, while others are pronounced when B cells and myeloid cells are in direct contact.

### B cells can abrogate the APC function of monocytes/macrophages and microglia—IL-10 is the key factor

**Fig. 3:**
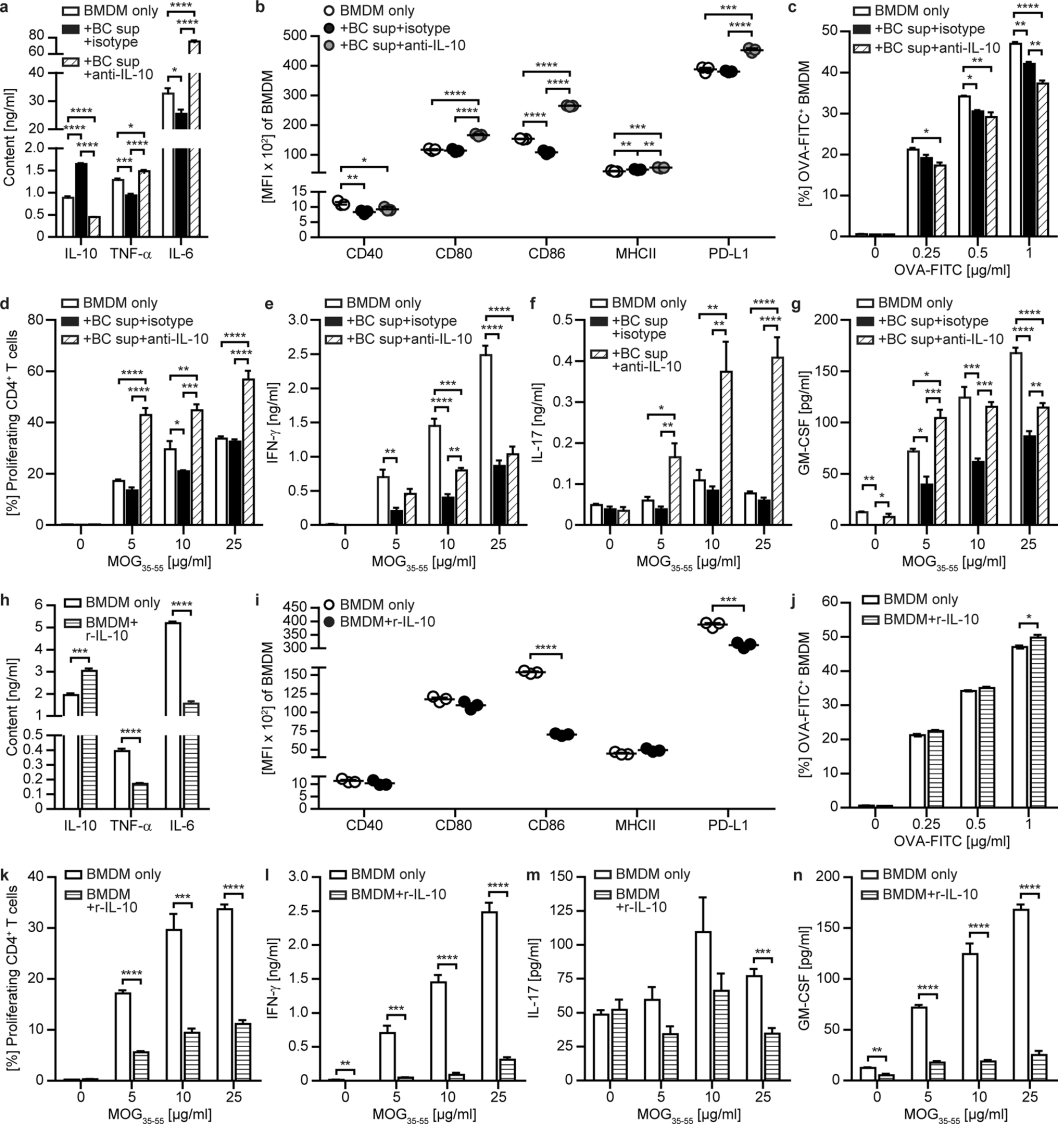
IL-10 regulates the inflammatory response of BMDM. Activated bone marrow-derived myeloid cells (BMDM) were either cultured alone (BMDM only), **a–g** with B cell supernatant (+ BC sup + isotype), B cell supernatant neutralized for IL-10 using IL-10 blocking antibodies (+ BC sup + anti-IL-10) or **h–n** with 1 ng/ml recombinant (r)IL-10 (BMDM + r-IL-10) for 48 h. After culture, **a**, **h** cytokine concentrations were determined in the supernatants via ELISA (*n* = 4–6 wells/condition) and **b**, **i** BMDM were analysed for activation and expression of molecules involved in antigen presentation shown as mean fluorescence intensity (MFI) using flow cytometry (*n* = 3 wells/condition). **c**, **j** Following pre-incubation with BC sup or r-IL-10, BMDM were cultured in the presence of FITC-labelled ovalbumin (OVA-FITC) for 2.5 h and the frequency ± of phagocytosing OVA-FITC^+^ cells (*n* = 3 wells/condition) was analysed via flow cytometry. **d–g**, **k–n** After pre-incubation with BC sup or r-IL-10, BMDM were co-cultured with carboxyfluorescein succinimidyl ester (CFSE)-stained MOG-specific T cells isolated from 2D2 mice in the presence of MOG peptide 35–55 (MOG_35-55_) for 72 h. **d**, **k** Frequency of proliferating CD4^+^ T cells (*n* = 4 wells/condition) was assessed via flow cytometry using CFSE dilution. **e–g**, **l–n** Cytokine concentrations were determined by ELISA (*n* = 4 wells/condition). The mean ± standard error of the mean is indicated in all graphs. Data sets are representative of at least 2–3 independent experiments. Asterisks indicate significant differences calculated using **a–g** one-way analysis of variance corrected by Holm-Sidak and **h–n** unpaired two-tailed *t*-test (*P ≤ 0.05, **P ≤ 0.01, ***P ≤ 0.001, ****P ≤ 0.0001)

To dissect which B cell properties mediate this regulatory effect on peripheral and CNS APC, we accordingly focused on B cell-secreted factors. At first, we screened the supernatant of B cells for secreted cytokines and chemokines. Besides pro-inflammatory IL-6, TNF-α, CC-chemokine ligand (CCL)2, CCL3 and CCL5, they released high amounts of anti-inflammatory IL-10 (Supplementary Fig. 2a, online resource). In EAE, IL-10 is known to dampen the activity of myeloid cells and to inhibit differentiation of CD4-positive T cells into pathogenic T helper (Th)1 and Th17 cells [11, 35]. Hence, we aimed to investigate whether B cell-secreted IL-10 was responsible for the observed altered phenotype of myeloid and CNS APC. For this purpose, we cultured BMDM together with cell supernatant harvested from activated B cells either containing all secreted cytokines or functionally neutralized for IL-10 (Supplementary Fig. 2b, online resource). In line with the results obtained for co-cultures of BMDM and B cells, IL-10-containing B cell supernatant significantly reduced the secretion of pro-inflammatory TNF-α and IL-6 (Fig. 3a; + BC sup + isotype) and downregulated the expression of CD40 and CD86 (Fig. 3b; + BC sup + isotype). In contrast, neutralizing IL-10 substantially enhanced the production of pro-inflammatory cytokines (Fig. 3a; + BC sup + anti-IL-10) and resulted in an increased expression of CD80, CD86 and PD-L1 (Fig. 3b; + BC sup + anti-IL-10). Of note, neutralizing IL-10 in B cell supernatant may also reduce the amount of detectable IL-10 secreted by BMDM in the culture setting.

In order to elucidate to what extent the phenotypical changes observed translate to functional alterations, we next assessed the ability of pre-incubated BMDM to phagocytose and to activate T cells when used as APC. Pre-incubation with B cell supernatant slightly reduced the overall phagocytosis rate of BMDM independent of IL-10 (Fig. 3c) and had no further effect on opsonization-enhanced phagocytosis (Supplementary Fig. 3a, online resource). In contrast, APC function of BMDM was altered markedly; pre-exposure of BMDM with IL-10-containing B cell supernatant significantly reduced their ability to activate T cells and to promote differentiation into encephalitogenic T cells (Fig. 3d–g; + BC sup + isotype). When BMDM were pre-exposed to B cell supernatant neutralized of IL-10, this regulatory effect was reversed and in part T cell activation was even boosted; specifically, we observed an enhanced T cell proliferation and Th17 differentiation (Fig. 3d–g; + BC sup + anti-IL-10), indeed pointing towards other soluble B cell-derived factors counteracting regulatory IL-10.

To control for these multiple factors and also to confirm the key regulatory function of IL-10 in our system, we also assessed the direct effect of recombinant (r)IL-10. Clearly, the addition of rIL-10 significantly reduced the secretion of pro-inflammatory cytokines, the expression of surface molecules on BMDM and the capacity of BMDM to induce T cell proliferation and differentiation, corroborating and confirming all the observations made by neutralizing IL-10 within B cell supernatants (Fig. 3h–n; Supplementary Fig. 3b, c, online resource). In summary, these data highlight that B cell-secreted IL-10 substantially alters the phenotype and function of myeloid cells, which results in a strongly reduced capacity to generate encephalitogenic T cells.

**Fig. 4:**
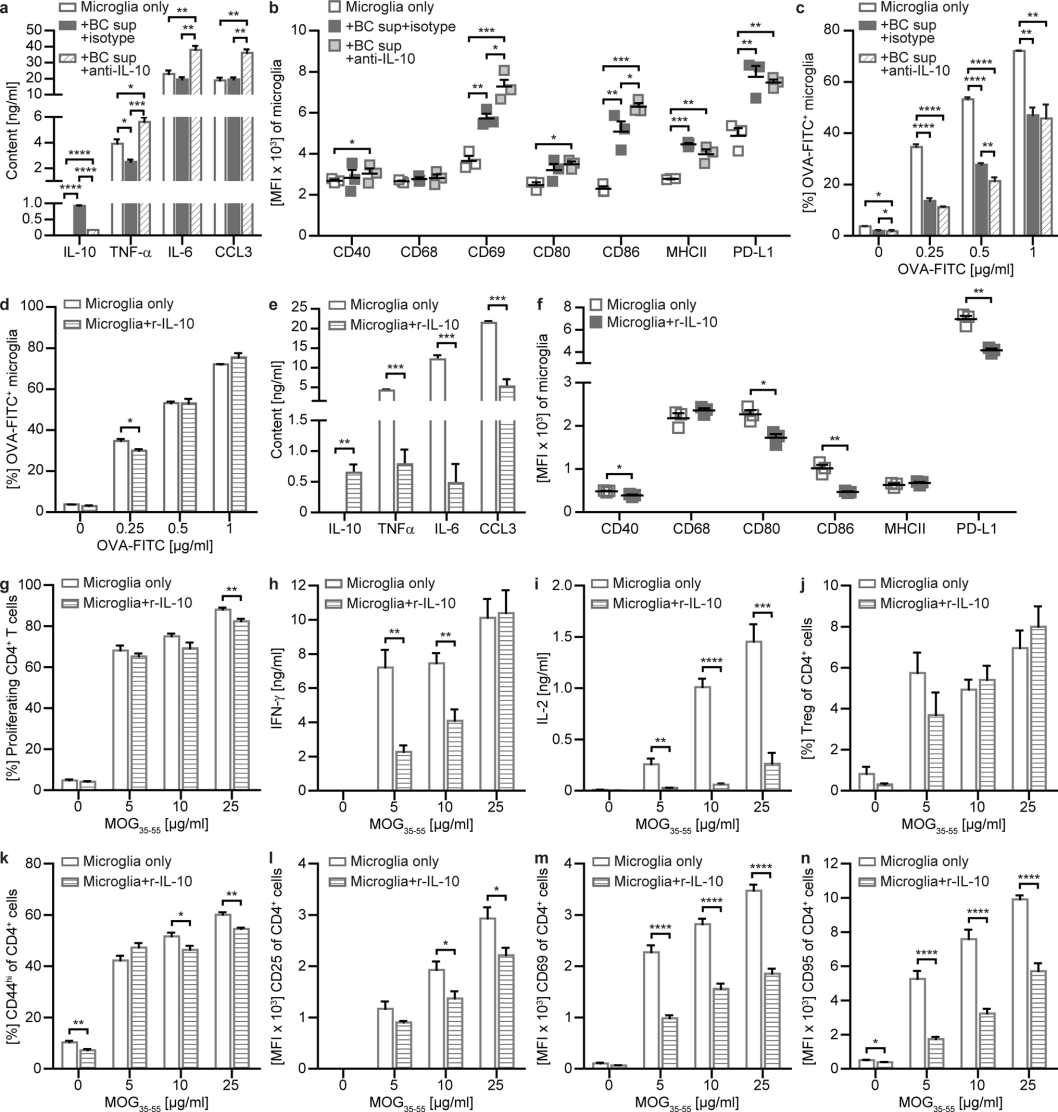
IL-10 modulates inflammatory properties of microglia. Activated primary microglia were either cultured alone (Microglia only), **a–c** with B cell supernatant (+ BC sup + isotype), B cell supernatant neutralized for IL-10 using IL-10 blocking antibodies (+ BC sup + anti-IL-10) or **d–n** with 1 ng/ml recombinant (r)IL-10 (Microglia + r-IL-10) for 24 h. After culture, **a**, **e** cytokine concentrations were determined by ELISA (*n* = 3 wells/condition) and **b**, **f** microglia were analysed for activation and expression of molecules involved in antigen presentation shown as mean fluorescence intensity (MFI) using flow cytometry (*n* = 3 wells/condition). **c**, **d** Following pre-incubation with BC sup or r-IL10, microglia were cultured in the presence of FITC-labelled ovalbumin (OVA-FITC) for 2.5 and the frequency of phagocytosing OVA-FITC^+^ cells (*n* = 3 wells/condition) was analysed via flow cytometry. **g–n** Following pre-incubation with r-IL-10, microglia were co-cultured with MOG-specific T cells isolated from 2D2 mice in the presence of MOG peptide 35–55 (MOG_35-55_) for 72 h. **g** Frequency of proliferating CD4^+^ T cells (*n* = 6 wells/condition) was measured by cell size using flow cytometry and **h**, **i** cytokine concentrations in cell supernatants were determined by ELISA (*n* = 6 wells/condition). **j** Frequency of FoxP3^+^ CD25^+^ regulatory T cells (Treg) and **k)** CD44^hi^ cells within CD4^+^ T cells (*n* = 4–6 wells/condition) as well as expression of **l** CD25, **m** CD69 and **n** CD95 of CD4^+^ T cells shown as mean fluorescence intensity (MFI) was analysed via flow cytometry (*n* = 4–6 wells/condition). The mean ± standard error of the mean is indicated in all graphs. Data sets are representative of at least three independent experiments. Asterisks indicate significant differences calculated using **a–c** one-way analysis of variance corrected by Holm-Sidak and **d–n** unpaired two-tailed *t*-test (*P ≤ 0.05, **P ≤ 0.01, ***P ≤ 0.001, ****P ≤ 0.0001)

To exert pathogenic effector function within the CNS, peripherally primed T cells need to be reactivated locally. In principle, this can be achieved by hematopoietic, CNS-infiltrating APC, such as macrophages or, alternatively by CNS resident APC. Among the later, microglia are assumed to be the key APC providing this mandatory second T cell activation signal [3]. Accordingly, we next analysed the effect of B cells and B cell-derived IL-10 on primary microglia. Paralleling our findings with BMDM, IL-10-containing B cell supernatant reduced the secretion of TNF-α, while blocking of IL-10 resulted in a significant increase in secreted TNF-α, IL-6 and CCL3 (Fig. 4a). Moreover, the addition of B cell supernatant substantially upregulated the expression of CD69, CD80, CD86, MHC class II and PD-L1 on microglia, which was further accelerated by the neutralization of IL-10 (Fig. 4b). Microglia furthermore displayed a reduced phagocytosis rate upon culture with B cell supernatant; however, this effect occurred largely independent of IL-10 (Fig. 4c).

In order to consolidate and mechanistically dissect these results, we again used rIL-10. Confirming the above described blocking experiment, the addition of rIL-10 to microglia had only minor effects on the capacity of these cells to phagocytose (Fig. 4d). Accordingly, we conclude that while B cells alter microglial phagocytosis, this must be mediated by factors other than IL-10. In contrast, rIL-10 strongly reduced the production of pro-inflammatory cytokines by microglia and downregulated the expression of activation markers and molecules involved in antigen presentation (Fig. 4e, f). To assess whether this may translate towards a change in function, pre-incubated microglia were again cultured with T cells in the presence of antigen. Although rIL-10 did not alter the capacity of microglia to promote overall T cell proliferation (Fig. 4g), T cell activation and differentiation were strongly impaired (Fig. 4h–n); specifically, T cells expressed substantially lower levels of CD25, CD69 and CD95 and secreted significantly lower amounts of pro-inflammatory interferon gamma (IFN-α and IL-2, suggesting that B cell-derived IL-10 effectively suppresses the capacity of microglia to (re-)activate encephalitogenic T cells. In conjunction, these results highlight that B cells and specifically B cell-derived IL-10 have the ability to regulate the pro-inflammatory APC function of myeloid cells, both in the periphery and within the CNS.

### Anti-CD20 exacerbates a T cell-mediated mouse model of MS, in which B cells are not involved in a pathogenic manner

**Fig. 5:**
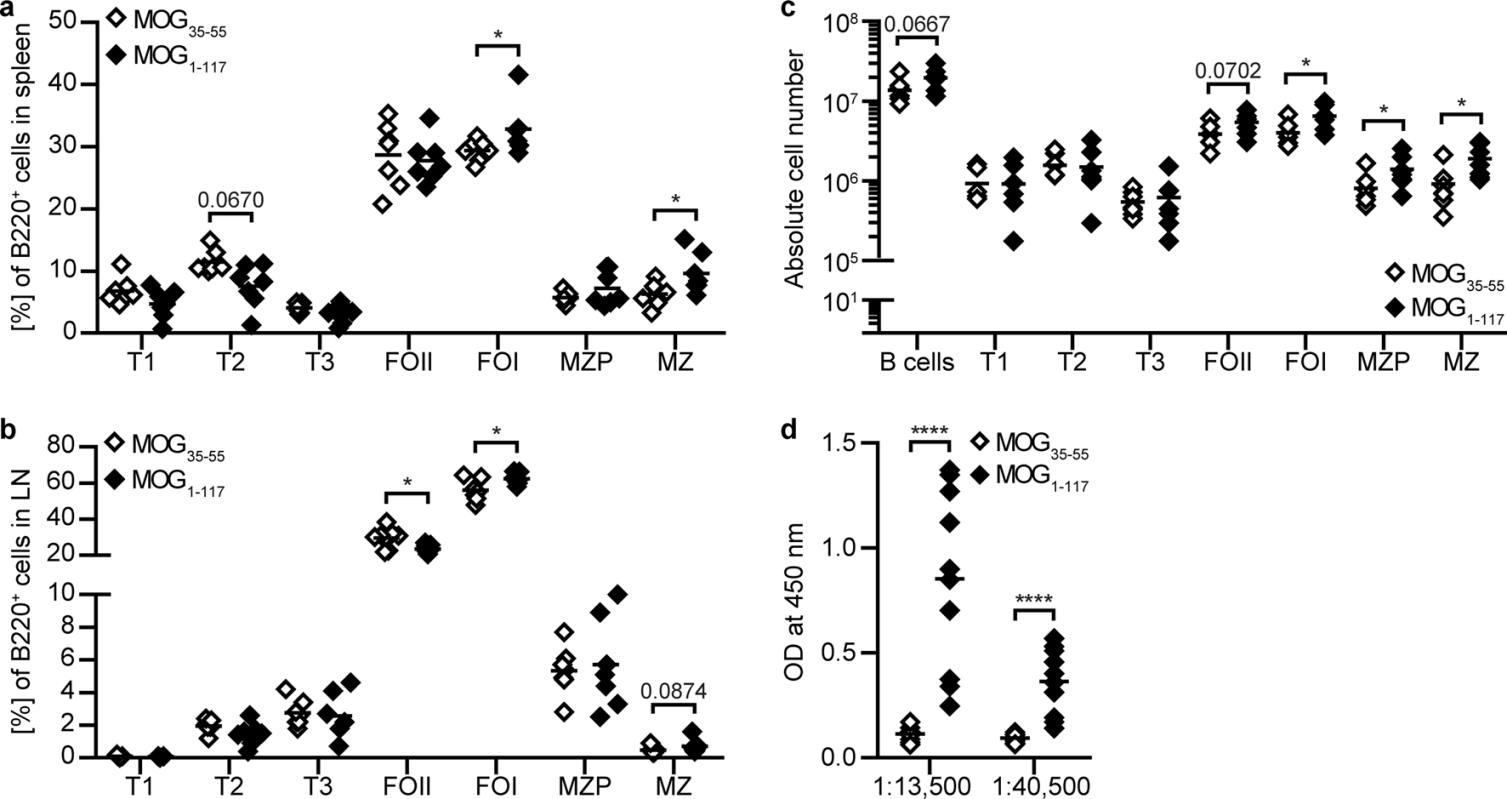
In MOG p35-55 versus MOG protein 1–117 EAE mouse model, B cells become less differentiated and produce decreased amounts of pathogenic MOG-specific antibodies. C57BL/6J mice were immunized with MOG peptide 35–55 (MOG_35-55_) or MOG protein 1–117 (MOG_1–117_). B cells from **a**, **c** spleen and **b** inguinal lymph nodes (LN), as well as **d** blood serum were isolated on day 24 post-immunization. **a–c** B cells (B220^+^) were categorized into transitional (T1: B220^+^CD93^+^IgM^+^; T2: B220^+^CD93^+^IgM^+^CD23^+^; T3: B220^+^CD93^+^CD23^+^), follicular (FOI: B220^+^CD19^+^IgD^+^; FOII: B220^+^CD19^+^IgD^+^IgM^+^), marginal zone precursor (MZP: B220^+^CD19^+^CD21^+^IgM^+^IgD^+^CD23^+^) and marginal zone (MZ: B220^+^CD19^+^CD21^+^IgM^+^IgD^+^) cells (*n* = 7). **d** Anti-MOG IgG serum levels were determined by ELISA at serum dilutions of 1:13,500 and 1:40,500 (*n* = 10). The median is indicated in all graphs. Data sets are representative of two independent experiments. Asterisks indicate significant difference calculated using the unpaired two-tailed Mann–Whitney *U* test (non-Gaussian distribution) or the unpaired two-tailed *t*-test (Gaussian distribution) (*P ≤ 0.05, ****P ≤ 0.0001)

Our in vitro studies elucidated that B cells, by provision of IL-10, regulate the pro-inflammatory activity of peripheral and CNS myeloid cells. In conjunction with our observation that anti-CD20 treatment is associated with an enhanced activity of blood monocytes in patients with MS, these findings highlight a regulatory axis between B cells and myeloid cells, which is collaterally abrogated by non-selective B cell depletion. To investigate this scenario in vivo, we established and utilized a mouse model of MS, in which the induction regimen does not activate B cells in an antigen-specific manner. We immunized mice with the T cell determinant of myelin oligodendrocyte glycoprotein (MOG) peptide (p)35–55 and compared this model to EAE induced by immunization with folded recombinant full-length MOG protein 1–117. As indicated in Fig. 5a–d, MOG protein immunization leads to an activation and differentiation of B cells, which is not observed after immunization with MOG p35-55. As a result, only MOG protein-immunized mice develop anti-MOG antibody titre [41] (Fig. 5d), while immunization with MOG p35-55 preferentially increased B cellular production of IL-10 (Supplementary Fig. 4, online resource).

**Fig. 6:**
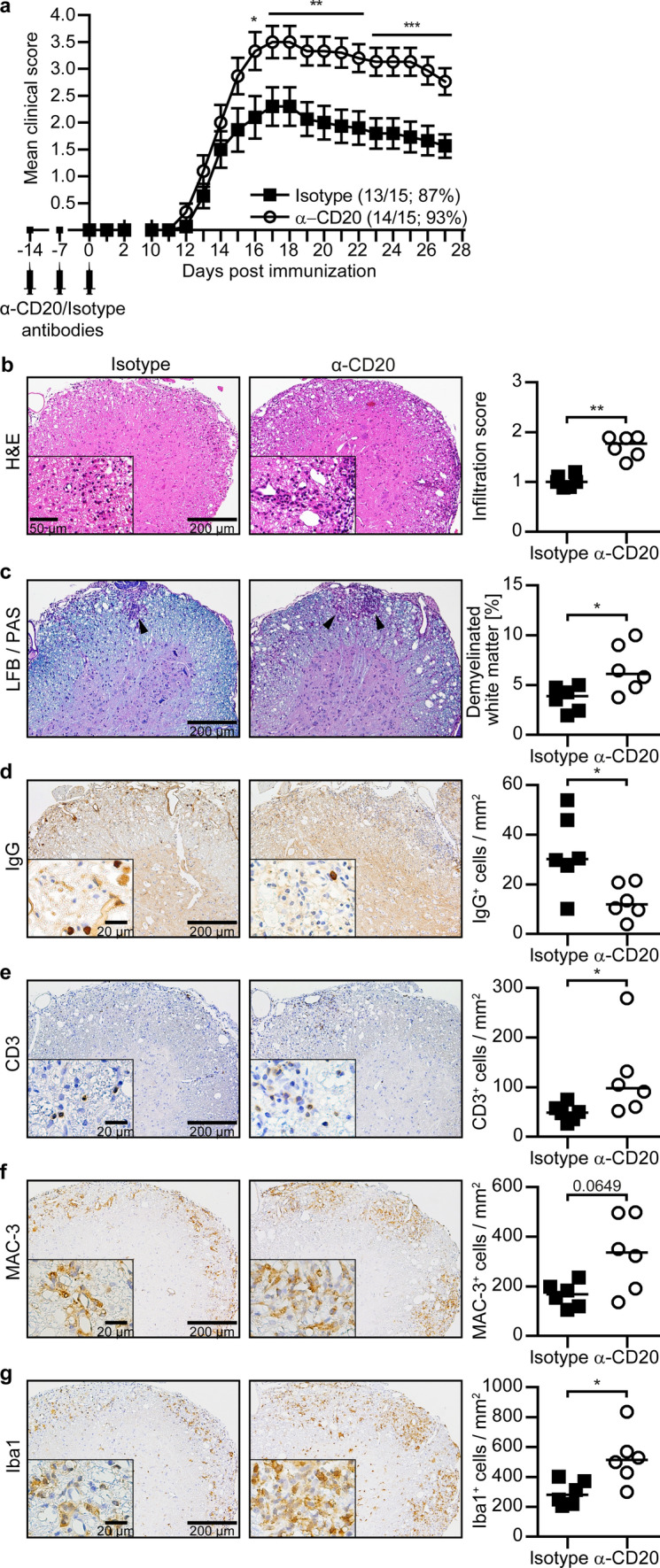
Depletion of B cells is associated with increased clinical severity, spinal cord infiltration and demyelination during MOG p35-55 EAE. C57BL/6 mice were treated weekly with 0.2 mg anti-CD20 (α-CD20) or isotype control antibodies for three consecutive weeks followed by immunization with MOG peptide 35–55. **a** Group EAE score with disease incidence indicated in brackets (*n* = 15). **b** Overall spinal cord inflammation was evaluated by haematoxylin and eosin (H&E) staining and assessed on a scale from 0 to 3 as follows: 0 = no infiltration; 1 = minor infiltration; 2 = moderate infiltration; 3 = pronounced infiltration. Representative sections (left) and inflammatory scores (right; *n* = 6). **c** Demyelinated white matter areas were assessed by luxol fast blue/periodic acid-Schiff (LFB/PAS) staining. Representative sections (left; black arrowheads indicate demyelinated white matter) and percentage of demyelinated white matter in relation to the total white matter area (right; *n* = 6). **d–g** Cellular CNS infiltration was assessed by immunohistochemical staining for **d** IgG, **e** CD3, **f** Mac-3 and **g** Iba1. Representative sections (left) and number of cells/mm^2^ per group (right; *n* = 6). In the graphs, **a** mean ± standard error of the mean (SEM) or **b–g** median is indicated. Data sets are representative of two independent experiments. Asterisks indicate significant difference calculated using the unpaired two-tailed Mann–Whitney *U* test (*P ≤ 0.05, **P ≤ 0.01, ***P ≤ 0.001)

Utilizing this model, mice were depleted of B cells using murine anti-CD20 antibodies prior to immunization. Upon MOG p35-55 immunization, B cell-depleted mice developed substantially aggravated EAE symptoms as indicated in Fig. 6a. Histologically, these results were supported by significantly higher levels of CNS inflammation and demyelination, as well as a higher number of CNS-infiltrating T cells and activated myeloid cells (Fig. 6b–g). These findings highlight that the clinical outcome of anti-CD20-mediated B cell depletion is determined by the pre-existing B cell phenotype and that depletion of B cells with preferential regulatory function translates to clinical deterioration.

### In vivo loss of B cell regulation is associated with an enhanced activity of pro-inflammatory monocytes, macrophages and microglia within the CNS

**Fig. 7:**
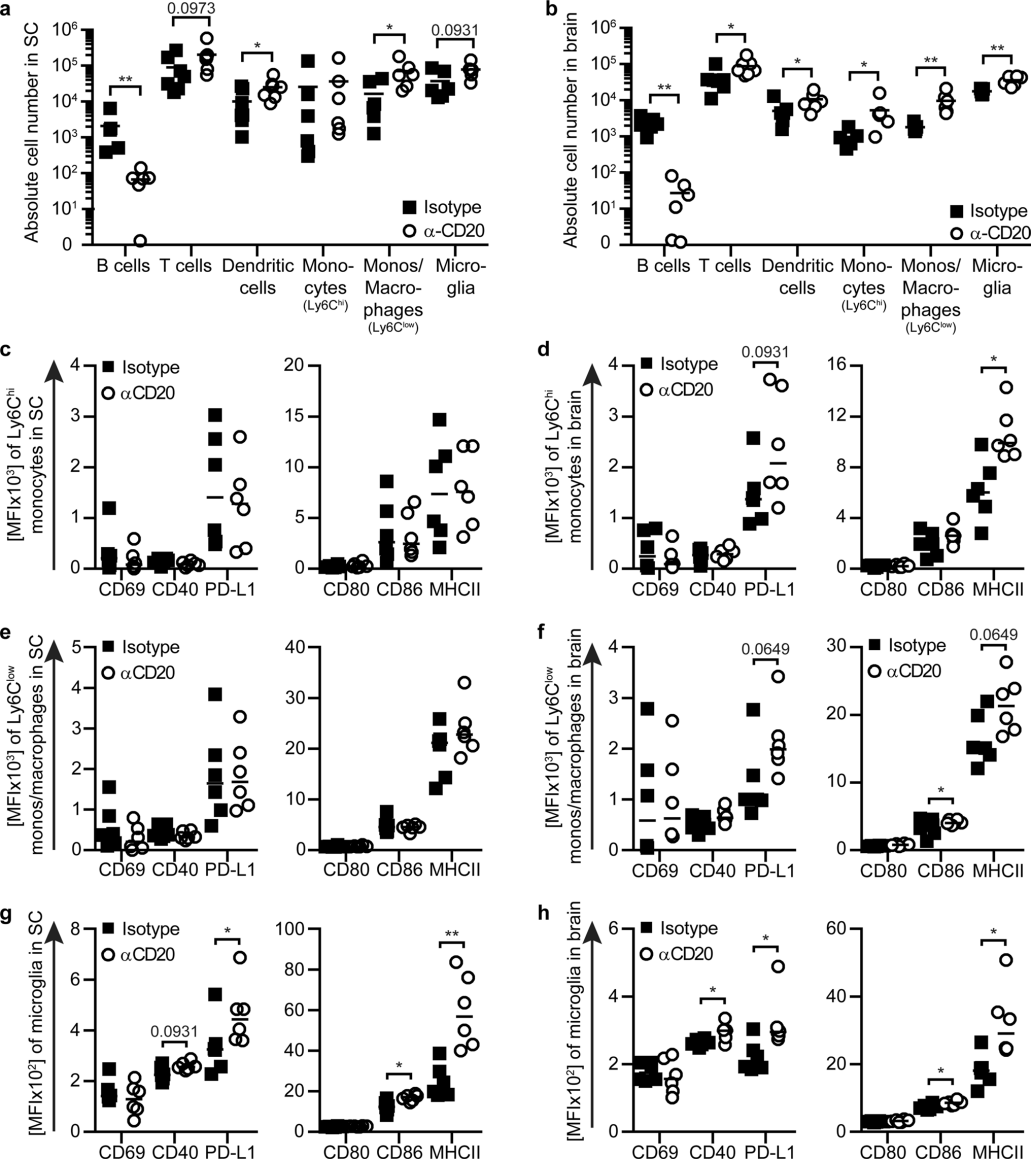
Depletion of B cells is associated with enhanced activation and up-regulation of molecules involved in antigen-presentation on monocytes, macrophages and microglia during EAE. Mice were treated weekly with 0.2 mg anti-CD20 (α-CD20) or isotype antibodies for three consecutive weeks followed by immunization with MOG peptide 35–55. Composition of infiltrating cells in **a** spinal cord (SC) and **b** brain (B cells: CD19^+^CD20^+^, T cells: CD3^+^, dendritic cells: CD11c^+^, monocytes (monos; Ly6C^hi^): CD11b^+^CD45^hi^Ly6C^hi^, monos/macrophages (Ly6C^low^): CD11b^+^CD45^hi^Ly6C^−^, microglia: CD11b^+^CD45^low^Ly6C^−^) were analysed by flow cytometry (*n* = 6–7). **c**, **d** Monocytes (Ly6C^hi^), **e**, **f** monos/macrophages (Ly6C^low^) and **g**, **h** microglia were isolated from **c**, **e**, **g** spinal cord (SC) and **d**, **f**, **h** brain. Activation and expression of molecules involved in antigen presentation were analysed by flow cytometry and are shown as mean fluorescence intensity (MFI; *n* = 6). The median is indicated in all graphs. Data sets are representative of two independent experiments. Asterisks indicate significant difference calculated using the unpaired two-tailed Mann–Whitney *U* test (*P ≤ 0.05, **P ≤ 0.01)

We next analysed whether these clinical results may be reflective of an unleashed pro-inflammatory activity of myeloid cells and examined CNS myeloid cells. We observed that the CNS of B cell-depleted mice contained higher absolute numbers of T cells, dendritic cells, monocytes/macrophages as well as activated microglia (Fig. 7a, b). When we characterized these cells in greater detail, we determined that both in the spinal cord and in the brain monocytes, macrophages and microglia showed an upregulation of activation markers and surface molecules involved in antigen presentation (Fig. 7c–h). In an attempt to address the question whether this regulatory B cell effect on myeloid cells had occurred in the periphery, and thus prior to CNS infiltration, or whether alternatively lack of B cell regulation within the CNS had unleashed myeloid cells within the CNS, we performed the similar study in established MOG p35-55-induced EAE at the peak of the disease. Of note, anti-CD20 applied at this later time-point, significantly lowered the number of CNS B cells, which resulted in an upregulated activity of CNS-established, hematopoietic myeloid cells as well as CNS resident microglia (Supplementary Fig. 5a–i, online resource). Furthermore, we conducted the equivalent study in naïve, non-immunized mice and observed no effect of anti-CD20 treatment on CNS myeloid cells (Supplementary Fig. 6a–f, online resource). Collectively, and in conjunction with our in vitro findings, these results highlight that B cells have the ability to regulate myeloid cells within the CNS, a property which is abrogated by systemic anti-CD20 treatment.

### Adoptive transfer of IL-10-providing B cells dampens the activity of CNS myeloid cells and restores B cell-mediated suppression of experimental MS

**Fig. 8:**
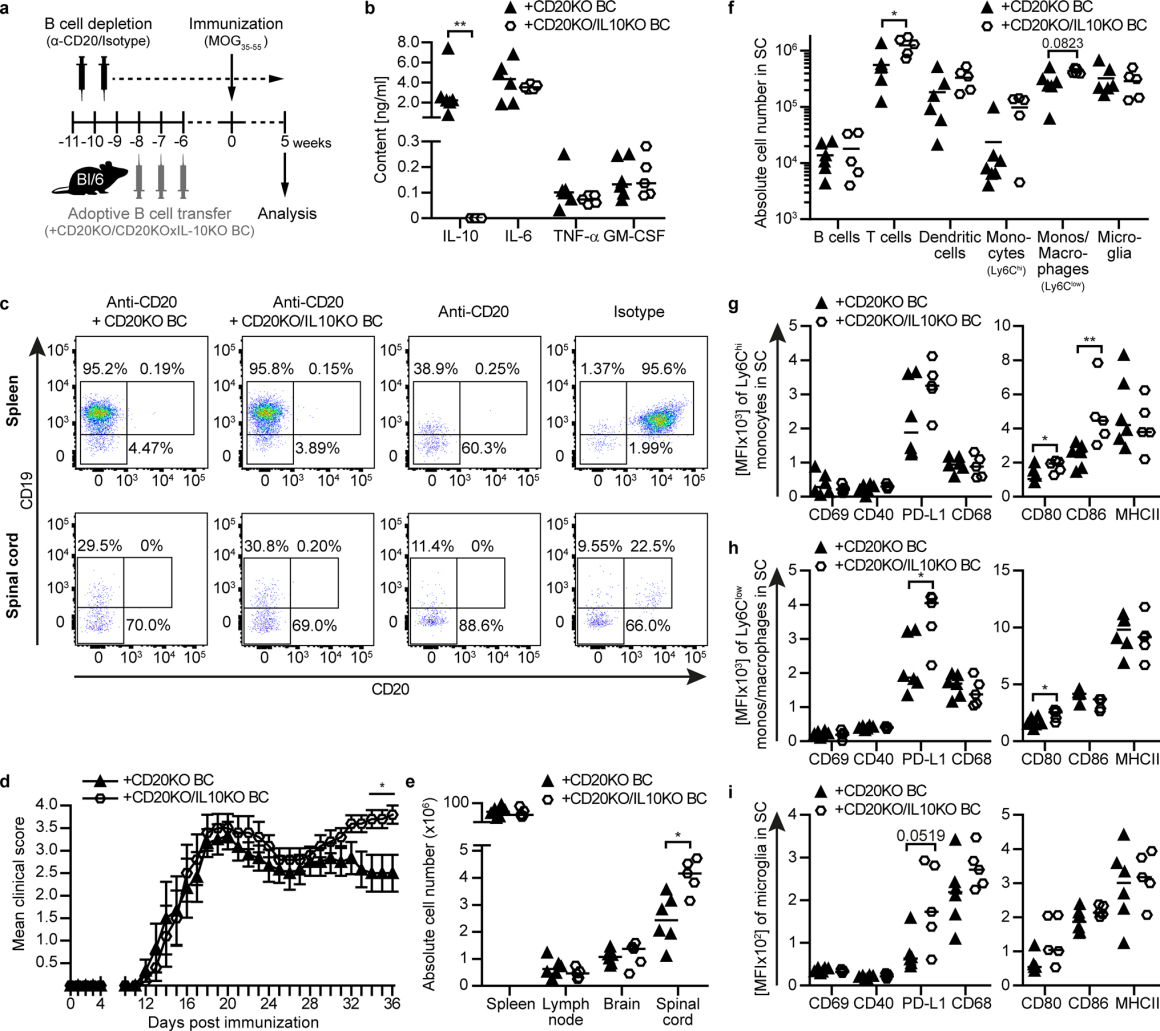
B cell-derived IL-10 is required to dampen activity of CNS-resident microglia to induce disease recovery. C57BL/6J mice received 0.2 mg anti-CD20 or isotype control antibody weekly throughout the whole experiment. After adoptive transfer of 30 × 10^6^ CD20 knockout (KO) or CD20KO/IL-10KO B cells, mice were immunized with MOG peptide 35–55. **a** Overview of experimental setup. **b** Week 5 after immunization, splenic B cells were purified, stimulated with 10 µg/ml CpG for 20 h and cytokine concentrations were determined via ELISA (*n* = 5–6). **c** B cell transfer was controlled by flow cytometry at the end of the experiment. **d** Group EAE score (*n* = 5–6). **e** Absolute numbers of immune cells in the spleen, inguinal lymph node, brain and spinal cord were quantified via microscopy (*n* = 5–6). **f** Composition of spinal cord (SC)-infiltrating cells (B cells: CD19^+^CD20^+^, T cells: CD3^+^, dendritic cells: CD11c^+^, monocytes (monos; Ly6C^hi^): CD11b^+^CD45^hi^Ly6C^hi^, monos/macrophages (Ly6C^low^): CD11b^+^CD45^hi^Ly6C^−^, microglia: CD11b^+^CD45^low^Ly6C^−^) was analysed by flow cytometry (*n* = 5–6). **g** Monocytes (Ly6C^hi^; CD11b^+^CD45^hi^Ly6C^hi^), **h** monos/macrophages (Ly6C^low^; CD11b^+^CD45^hi^Ly6C^−^) and **i** microglia (CD11b^+^CD45^low^Ly6C^−^) were isolated from spinal cord (SC). Activation and expression of molecules involved in antigen presentation were analysed by flow cytometry and are shown as mean fluorescence intensity (MFI; *n* = 5–6). In the graphs, **b**, **e-i** median or **d** mean ± standard error of the mean (SEM) is indicated. All data sets are representative of two independent experiments. Asterisks indicate significant difference calculated using the unpaired two-tailed Mann–Whitney *U* test (*P ≤ 0.05, **P ≤ 0.01)

In a last set of experiments, we assessed whether aggravation of EAE in the absence of B cells can be ascribed to the loss of B cell-secreted IL-10. For this purpose, we generated a model, in which B cells can produce IL-10 or genetically lack to do so (Fig. 8a, b). Specifically, we depleted B cells of naïve mice using anti-CD20 antibodies and subsequently reconstituted these animals with B cells secreting normal amounts of cytokines, or with IL-10 knockout (KO) B cells that lack the ability to produce IL-10. In all of these adoptive transfer experiments, CD20 KO B cells were used, in order to make transferred B cells resistant to circulating anti-CD20. Importantly, adoptively transferred B cells efficiently repopulated the spleen in recipient mice and later on infiltrated the spinal cord during EAE (Fig. 8c). Strikingly, adoptive transfer of IL-10 competent B cells restored recovery after acute EAE, while recipients of IL-10 KO B cells failed to recover and instead further deteriorated in the chronic phase (Fig. 8d). As in fully B cell-depleted mice, this deterioration was associated with an enhanced immune cell infiltration into the CNS, as well as with an elevated activity level of monocytes/macrophages and microglia within the CNS (Fig. 8e–i). These data corroborate that by provision of IL-10, B cells have the ability to control the activity of CNS myeloid cells, which permits recovery from acute EAE. In perspective, these findings highlight that B cells or B cell subsets exert a clinically relevant anti-inflammatory function within the chronically inflamed CNS, a property which may be desirable to maintain or even foster in chronic progression of CNS autoimmune disease.

## Discussion

We started our investigation on a possible regulatory role of B cells in MS by the simple observation that anti-CD20-mediated depletion of B cells, a highly effective therapy to prevent acute relapses, is associated with an enhanced pro-inflammatory activity of blood monocytes [20]. Of note, this effect occurred regularly and frequently in individual patients undergoing anti-CD20 treatment. In parallel, we assessed the ability of B cells from these patients to produce regulatory factors, such as anti-inflammatory IL-10. We observed that various CD20-positive B cell subsets can produce anti-inflammatory cytokines and, importantly, the respective ability to do so did not differ from healthy individuals. These findings generated the hypothesis that in patients with MS B cells can control the activity of peripheral and CNS myeloid cells and that this regulatory axis is extinguished by anti-CD20.

In an approach to assess the functional impact of this observation, we determined that the presence of B cells or the supernatant from activated B cells indeed limits the ability of macrophages to be activated in a pro-inflammatory manner. Functionally blocking IL-10 in this system identified this B cell-secreted cytokine as key factor. Performing the identical experiments with microglia generated similar results, highlighting that B cells broadly shape cells of myeloid origin and that B cell regulation may similarly be of relevance within the chronically inflamed CNS. Based on these results, we generated an in vivo setting to study B cell regulation. In the absence of pathogenic B cell function, preventative depletion of B cells via anti-CD20 substantially exacerbated subsequent experimental MS. This deterioration was associated with an unleased activity of macrophages as well as microglia within the CNS. Of note, this enhancement of CNS myeloid cell function similarly occurred when anti-CD20 was initiated in established EAE. Adoptive transfer of B cells back into depleted mice reversed both the clinical deterioration and regulation of CNS macrophages and microglia, while B cells incapable of producing IL-10 failed to do so. In summary, these findings highlight that B cell regulation persists in chronic CNS inflammation and that abrogation of this regulatory axis propagates CNS autoimmune disease via an unleased activity of CNS myeloid cells.

These findings have several implications; in line with other studies [14, 22], we identified that the identical B cell population can produce pro- as well as anti-inflammatory factors, strictly depending on the stimulus, the respective cellular milieu and largely independent of the B cell differentiation status. This may indicate that the earlier concept of a cellular dichotomy (regulatory vs. pathogenic effector B cells) [8] may need to be revisited, and that instead, a gradual description of regulatory versus pathogenic B cell properties might be more accurate [36].

This revised concept generates new perspectives on the role of B cells in various inflammatory conditions including MS. Thus far, the sole presence of immune cells including B cells within the CNS is associated with pathogenic function. In this regard, a better characterization of CNS B cells and plasma cells, including their spatial relation with CNS-located myeloid cells, is required and possibly on the horizon due to novel powerful microscopy and transcriptomic tools [9].

One central finding of our study is that B cells control the activity of cells of myeloid origin and that pan B cell depletion via anti-CD20 extinguishes this regulatory axis. Hereby, our findings critically complement our knowledge on the immunological consequences of widely used anti-CD20 antibodies. Clearly, a respective clinical impact of this observation may primarily depend on the underlying condition. In MS, however, activated CNS-infiltrated macrophages and microglia are assumed to be key players driving chronic progression within the CNS. In our experimental study, anti-CD20-mediated depletion of B cells activated macrophages and microglia within the CNS, which exacerbated EAE in a model in which B cells are not involved pathogenically. In conjunction, these findings may suggest that while anti-CD20 most effectively prevents de novo CNS immune cell infiltration, lesion formation and thereby clinical MS relapses, it may not be a suitable treatment for the core process of progression independent of focal activity.

## Supplementary Information

Below is the link to the electronic supplementary material.

Supplementary file1 (DOCX 1350 kb)

## Data Availability

All data associated with this study are included in the main figures or the supplementary figures/tables (online resource).
