# Supplementary material for: IL-10-providing B cells govern pro-inflammatory activity of macrophages and microglia in CNS autoimmunity
Source: Acta Neuropathol. 2023 Mar 1;145(4):461–77. doi: 10.1007/s00401-023-02552-6 (PMC10020302; doi:10.1007/s00401-023-02552-6)
Supplement: Supplementary file 1 — Supplementary file1 (DOCX 1350 kb) [file 401_2023_2552_MOESM1_ESM.docx]

**IL-10-providing B cells govern pro-inflammatory activity of macrophages and microglia in CNS autoimmunity**

Anastasia Geladaris^1,2#^, Silke Häusser-Kinzel^1#^, Roxanne Pretzsch^1,3^, Nitzan Nissimov^1,4^, Klaus Lehmann-Horn^5^, Darius Häusler^1,2#^, Martin S. Weber^1,2,3#*^

^1^Institute of Neuropathology, University Medical Centre, Göttingen, Germany; ^2^Fraunhofer-Institute for Translational Medicine and Pharmacology ITMP, Göttingen, Germany; ^3^Department of Neurology, University Medical Centre, Göttingen, Germany; ^4^Department of Neurosurgery, Charité-Universitätsmedizin Berlin Corporate Member of Freie Universität Berlin and Humboldt-Universität zu Berlin, Germany; ^5^Department of Neurology, School of Medicine, Technical University of Munich, Germany;

# Anastasia Geladaris and Silke Häusser-Kinzel are co-first, Darius Häusler and Martin S. Weber are co-last authors.

*Address correspondence to: Martin S. Weber, MD

Department of Neuropathology

Department of Neurology

University Medical Center, Georg August University

Robert-Koch-Str. 40, 37099 Göttingen, Germany

Phone +49-551 39-7706
Fax +49-551-39-10800
E-mail: [martin.weber@med.uni-goettingen.de](mailto:martin.weber@med.uni-goettingen.de)

**Supplementary figures**


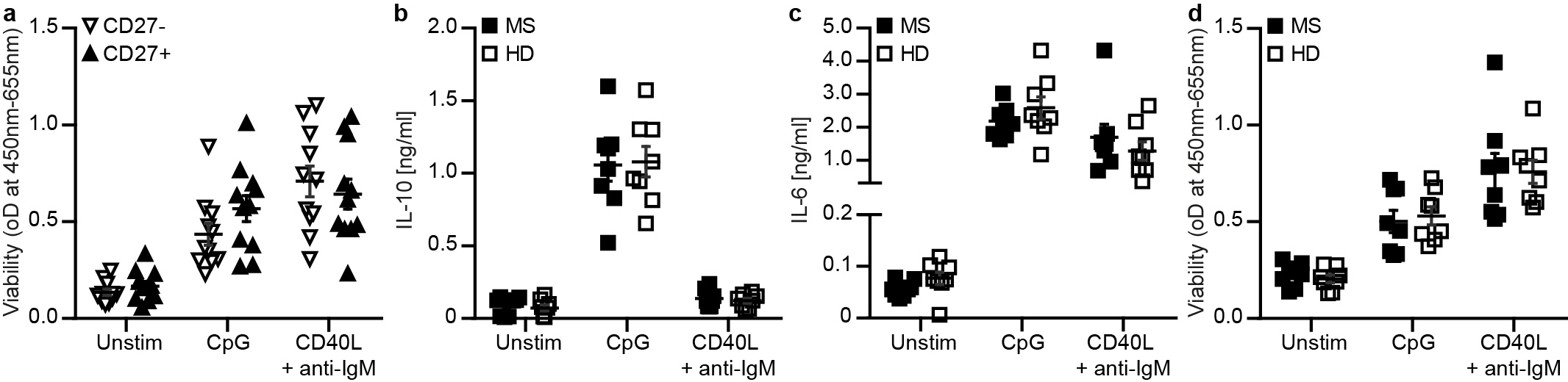


**Supplementary Fig. 1: B cells isolated from patients with MS and healthy controls secrete comparable amounts of IL-10 and IL‑6. a)** CD27^-^ and CD27^+^ B cells isolated from peripheral blood mononuclear cells (PBMC) of healthy donors (*n* = 11) were cultured without stimulation (unstim), stimulated with CpG (4 µg/ml) or stimulated with CD40 ligand (CD40L; 1 µg/ml) and anti-IgM antibody (40 µg/ml) for 48 hours. Viability of the cells was determined using colorimetric WST‑1 assay. **b-d)** B cells isolated from PBMC of patients with multiple sclerosis (MS; *n* = 8) and healthy donors (HD; *n* = 11) were cultured without stimulation (unstim), stimulated with CpG (4 µg/ml) or stimulated with CD40 ligand (CD40L; 1 µg/ml) and anti-IgM antibody (40 µg/ml) for 48 hours. Concentrations of secreted **b)** IL-10 and **c)** IL-6 were determined using ELISA and **d)** viability using colorimetric WST-1 assay. The mean ± standard error of the mean is indicated in all graphs.

| 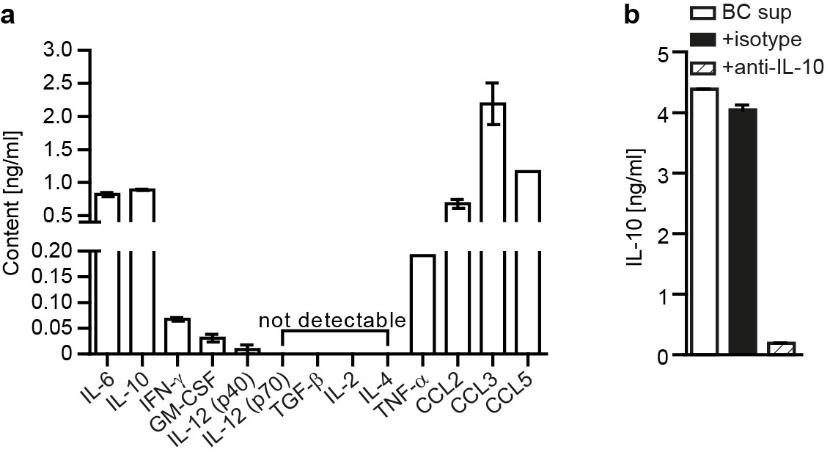 | **Supplementary Fig. 2: B cell cytokine profile and neutralization of IL-10 via anti-IL-10 antibodies.** Splenic B cells were isolated from C57BL/6J mice, stimulated with 5 µg/ml lipopolysaccharide and supernatants were collected after **a)** 24 or **b)** 48 hours. **a)** Indicated cytokines in the supernatant were determined using ELISA (*n* = 2) **b)** IL-10-neutralizing (anti-IL-10) or isotype control antibodies were added to the B cell supernatant for 20 minutes and IL-10 concentration was determined via ELISA. All data are shown as mean ± standard error of the mean. |
| --- | --- |

| **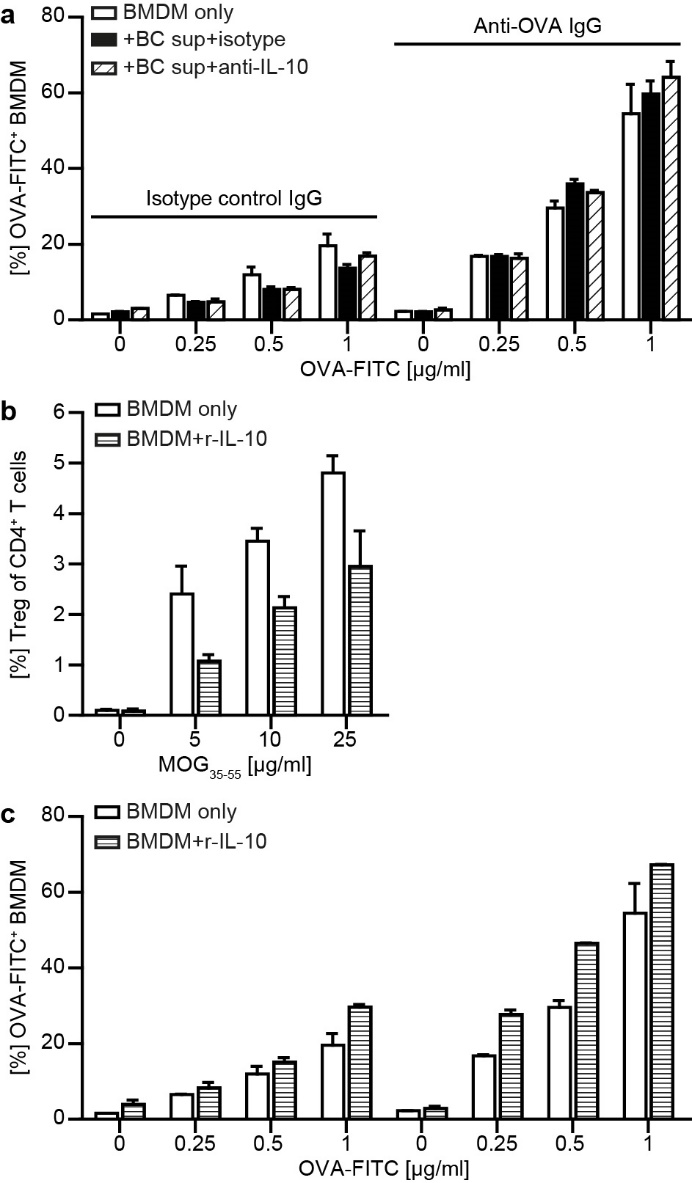** | **Supplementary 3: IL-10 exposure of BMDM neither influences opsonization-mediated phagocytosis nor development of regulatory T cells.** Activated bone marrow-derived myeloid cells (BMDM) were either cultured alone (BMDM only), **a)** with B cell supernatant (+BC sup+isotype), B cell supernatant neutralized for IL-10 using IL-10 blocking antibodies (+BC sup+anti-IL-10) or **b, c)** with 1 ng/ml recombinant (r)-IL-10 (BMDM+r-IL-10) for 48 hours. **a, c)** Following pre-incubation with BC sup or r-IL-10, BMDM were cultured with FITC-labelled ovalbumin (OVA-FITC) in the presence of anti-OVA antibody or an isotype control antibody (IgG) for 2.5 hours. Frequency of phagocytosing OVA-FITC^+^ cells (*n* = 2 wells/condition) was analysed via flow cytometry. **b)** After pre-incubation with r-IL-10, BMDM were co-cultured with carboxyfluorescein succinimidyl ester (CFSE)-stained MOG-specific T cells isolated from 2D2 mice in the presence of MOG peptide 35-55 for 72 hours. Frequency of CD25^+^ FoxP3^+^ regulatory T cells (Treg; *n* = 4 wells/condition) within CD4^+^ cells assessed via flow cytometry. All data are shown as mean ± standard error of the mean. |
| --- | --- |


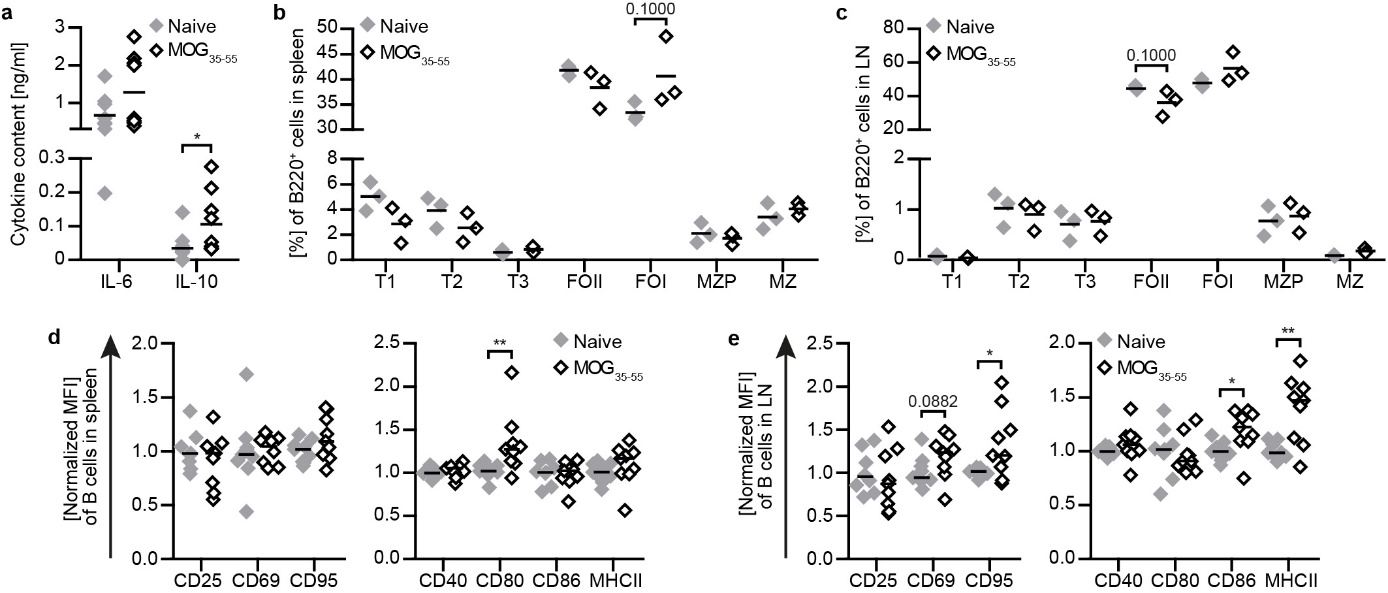


**Supplementary Fig. 4: MOG p35-55 immunization increases IL-10 production by B cells.** Naïve C57BL/6J mice or mice immunized with MOG peptide 35-55 (MOG_35-55_) were analysed on day 10 post-immunization. **a)** Splenic B cells were purified and stimulated with LPS [5 µg/ml] for 24 hours. Cytokine concentrations in the supernatants were determined via ELISA (*n* = 9; pooled from three independent experiments). B cells (B220^+^) from **b)** spleen and **c)** lymph nodes were categorized into transitional (T1: B220^+^CD93^+^IgM^+^; T2: B220^+^CD93^+^IgM^+^CD23^+^; T3: B220^+^CD93^+^CD23^+^), follicular (FOI: B220^+^CD19^+^IgD^+^; FOII: B220^+^CD19^+^IgD^+^IgM^+^), marginal zone precursor (MZP: B220^+^CD19^+^CD21^+^IgM^+^IgD^+^CD23^+^) and marginal zone (MZ: B220^+^CD19^+^CD21^+^IgM^+^IgD^+^) cells. Mean B cells from **d)** spleen and **e)** lymph nodes were analysed for activation and expression of molecules involved in antigen presentation shown as mean fluorescence intensity (MFI) using flow cytometry (*n* = 9; pooled from three independent experiments). The median is indicated in all graphs. Asterisks indicate significant difference calculated using the unpaired two-tailed Mann Whitney U-test (**P* ≤ 0.05, ***P* ≤ 0.01).


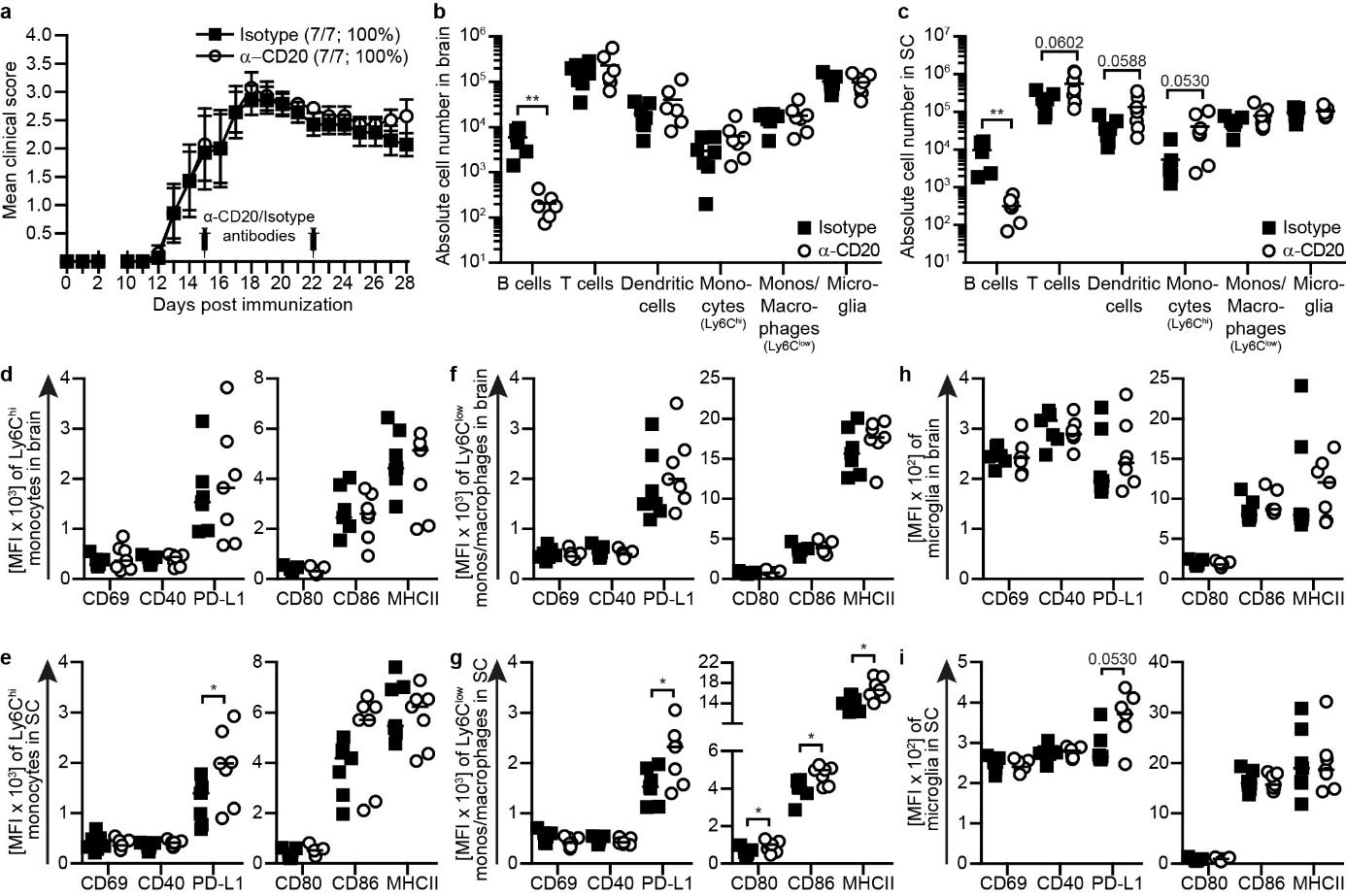


**Supplementary data Fig. 5: Depletion of B cells during the acute phase of the disease is associated with increased spinal cord infiltration, enhanced activation and expression of molecules involved in antigen presentation on myeloid cells.** C57BL/6 mice were treated with 0.2 mg anti-CD20 (α-CD20) or isotype antibodies on day 15 and 22 post immunization with MOG peptide 35-55. **a)** Group EAE score and disease incidence indicated in brackets (*n* = 7). Composition of CNS-infiltrating cells (B cells: CD19^+^CD20^+^, T cells: CD3^+^, dendritic cells: CD11c^+^, monocytes (monos; Ly6C^hi^): CD11b^+^CD45^hi^Ly6C^hi^, monos/macrophages (Ly6C^low^): CD11b^+^CD45^hi^Ly6C^-^, microglia: CD11b^+^CD45^low^Ly6C^-^) was analysed by flow cytometry in **b)** brain and **c)** spinal cord (SC) by flow cytometry (*n* = 6-7). **d, e)** Monocytes (Ly6C^hi^; CD11b^+^CD45^hi^Ly6C^hi^), **f, g)** monos/macrophages (Ly6C^low^; CD11b^+^CD45^hi^Ly6C^-^) and **h, i)** microglia (CD11b^+^CD45^low^Ly6C^-^) were isolated from **d, f, h**) brain or **e, g, i)** spinal cord (SC). Activation and expression of molecules involved in antigen presentation were analysed by flow cytometry are shown as mean fluorescence intensity (MFI; *n* = 7). In the graphs, **a)** mean ± standard error of the mean or **b-i)** median are indicated. Data sets are representative of two independent experiments. Asterisks indicate significant difference calculated using the unpaired two-tailed Mann Whitney U-test (**P* ≤ 0.05, ***P* ≤ 0.01).

**
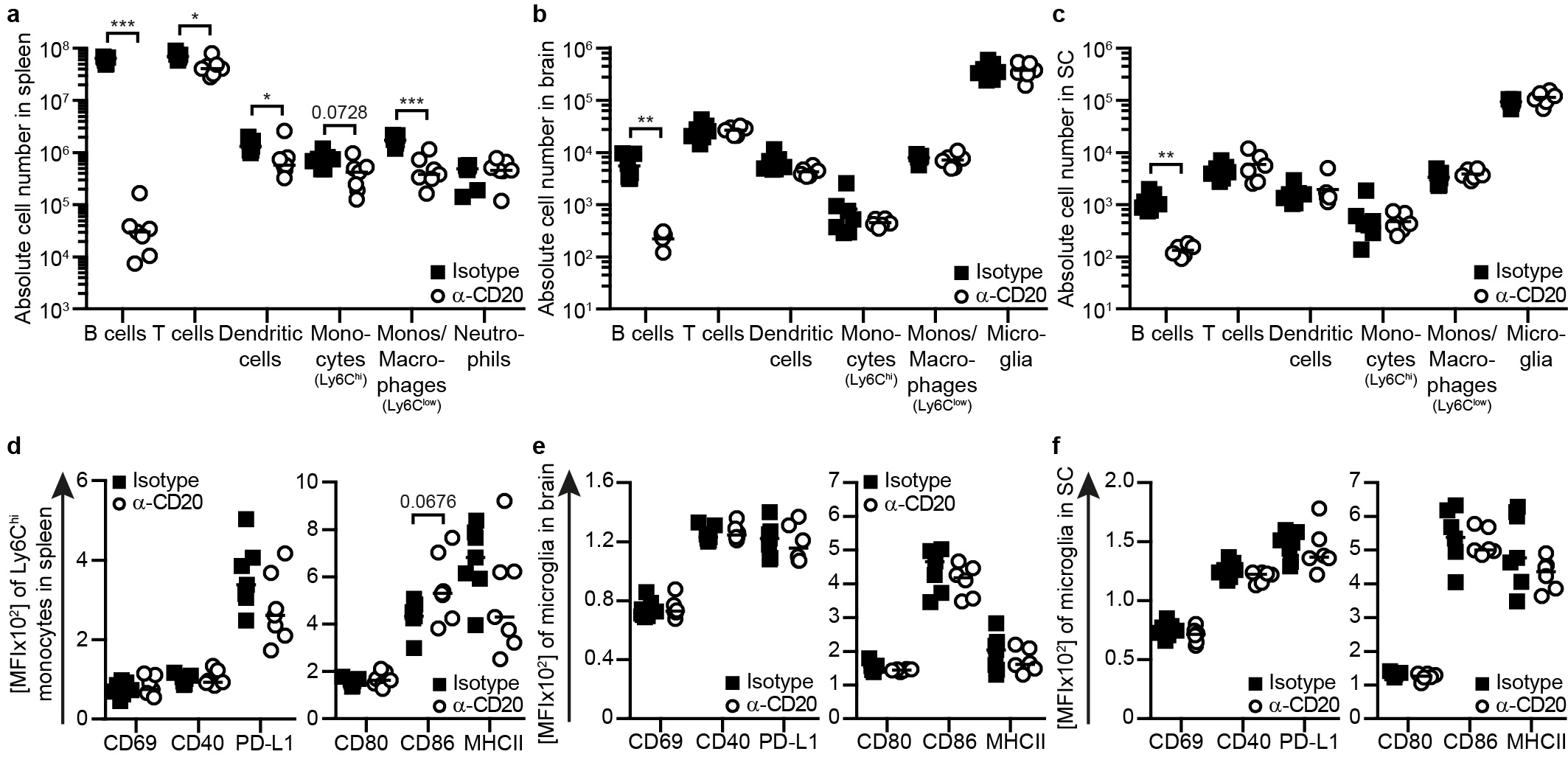
**

**Supplementary Fig. 6: B cell depletion has no effect on microglia cells in naïve mice.** Mice were treated weekly with 0.2 mg anti-CD20 (α-CD20) or isotype control antibodies for five consecutive weeks. Composition of immune cells in **a)** spleen (B cells: CD19^+^CD20^+^, T cells: CD3^+^, dendritic cells: CD11c^+^, monocytes (monos; Ly6C^hi^): CD11b^+^CD45^hi^Ly6C^hi^, monos/macrophages (Ly6C^low^): CD11b^+^CD45^hi^Ly6C^-^, neutrophils: CD11b^+^CD45^hi^Ly6G^+^Ly6C^int^), **b)** brain and **c)** spinal cord (SC; B cells: CD19^+^CD20^+^, T cells: CD3^+^, dendritic cells: CD11c^+^, monocytes (monos; Ly6C^hi^): CD11b^+^CD45^hi^Ly6C^hi^, monos/macrophages (Ly6C^low^): CD11b^+^CD45^hi^Ly6C^-^, microglia: CD11b^+^CD45^low^Ly6C^-^) were analysed by flow cytometry (*n* = 6-7). **d)** Ly6C^hi^ monocytes were isolated from spleen and microglia (CD11b^+^CD45^low^Ly6C^-^) were isolated from **e)** brain and **f)** spinal cord (SC). Activation and expression of molecules involved in antigen presentation were analysed by flow cytometry and are shown mean fluorescence intensity (MFI; *n* = 6-7). The median is indicated in all graphs. Data sets are representative of two independent experiments. Asterisks indicate significant difference calculated using the unpaired two-tailed Mann Whitney U-test (**P* ≤ 0.05, ***P* ≤ 0.01, ****P* ≤ 0.001).

*Supplementary table 1: Characteristics of subjects enrolled in studies on B cells*

|  | HC (Fig. 1a, b) | MS vs. HC (Extended data fig. 1b-d) | |
| --- | --- | --- | --- |
|  | HC (*n* = 11) | HC (*n* = 8) | MS (*n* = 8) |
| Age Mean ± SD | 27 ± 5.73 | 45 ± 15.09 | 45 ± 16.87 |
| Female/male | 10/1 | 4/4 | 4/4 |
| Time since diagnosis [months]; mean ± SD | n.a. | n.a. | 77.14 ± 75.71 |
| EDSS, mean ± SD | n.a. | n.a. | 2.86 ± 1.49 |
| Clinical course of MS [number] |  |  |  |
| RR-MS | n.a. | n.a. | 5 |
| SP-MS | n.a. | n.a. | 1 |
| PP-MS | n.a. | n.a. | 2 |
| Treatment |  |  |  |
| Corticosteroids (within last 2 months) | n.a. | n.a. | RR-MS: 2 |
| Glatiramer acetate (> 7 days, within last 2 months) | n.a. | n.a. | RR-MS: 2 |
| no treatment for > 1 year | n.a. | n.a. | RR-MS:1  SP-MS: 1  PP-MS: 2 |

HC = healthy control; MS = multiple sclerosis; SD = standard deviation; n. a. = not applicable; EDSS = Expanded Disability Status Scale; RR = relapsing-remitting; SP = secondary progressive; PP = primary progressive; DMD = disease modifying drug

*Supplementary table 2: Characteristics of patients enrolled in studies on CD14^+^ cells (Figure 1c-e)*

|  | Flow cytometry (*n* = 14) | ELISpot (*n* = 17) |
| --- | --- | --- |
| Age [years]; mean ± SD | 34.71 ± 8.30 | 36.00 ± 10.20 |
| Time since diagnosis [years]; mean ± SD | 8.86 ± 7.77 | 10.57 ± 9.15 |
| Female/male | 7/7 | 9/8 |
| EDSS; mean ± SD | 3.3 ± 2.01 | 3.5 ± 1.85 |
| Clinical course of MS [number] |  |  |
| RR-MS | 12 | 14 |
| SP-MS | 1 | 2 |
| CIS | 1 | 1 |
| Last treatment before rituximab |  |  |
| Azathioprine | RR-MS: 1 | RR-MS: 1 |
| Dimethyl fumarate | RR-MS: 2 | RR-MS: 2 |
| Fingolimod | RR-MS: 6 | RR-MS: 6 |
| Glatiramer acetate | RR-MS: 1 | RR-MS: 1 |
| Natalizumab | RR-MS: 1  SP-MS: 1 | RR-MS: 1  SP-MS: 1 |
| no treatment | RR-MS: 1  CIS: 1 | RR-MS: 3  SP-MS: 1  CIS: 1 |

SD = standard deviation; EDSS = Expanded Disability Status Scale; MS = multiple sclerosis; RR = relapsing-remitting; SP = secondary progressive; CIS = clinically isolated syndrome
